# Gold Nanoparticle-Modified Carbon-Fiber Microelectrodes for the Electrochemical Detection of Cd^2+^ via Fast-Scan Cyclic Voltammetry

**DOI:** 10.3390/mi15030294

**Published:** 2024-02-21

**Authors:** Noel Manring, Miriam Strini, Gene Koifman, Jessica L. Smeltz, Pavithra Pathirathna

**Affiliations:** Department of Chemistry and Chemical Engineering, Florida Institute of Technology, 150 W. University Blvd, Melbourne, FL 32901, USA; nmanring2020@my.fit.edu (N.M.); mstrini2021@my.fit.edu (M.S.); gkoifman2021@my.fit.edu (G.K.); jsmeltz@fit.edu (J.L.S.)

**Keywords:** carbon carbon-fiber microelectrodes, cadmium, fast scan cyclic voltammetry, gold nanoparticles, electrodeposition, real-time analysis

## Abstract

Neurotoxic heavy metals, such as Cd^2+^, pose a significant global health concern due to their increased environmental contamination and subsequent detrimental health hazards they pose to human beings. These metal ions can breach the blood–brain barrier, leading to severe and often irreversible damage to the central nervous system and other vital organs. Therefore, developing a highly sensitive, robust, and rapid in vivo detection method for these hazardous heavy metal ions is of the utmost importance for early detection, thus initiating timely therapeutics. Detecting ultra-low levels of toxic metal ions in vivo and obtaining accurate speciation information remains a challenge with conventional analytical techniques. In this study, we fabricated a novel carbon carbon-fiber microelectrode (CFM)-based sensor that can detect Cd^2+^ ions using fast-scan cyclic voltammetry by electrodepositing gold nanoparticles (AuNP). We optimized electrochemical parameters that generate a unique cyclic voltammogram (CV) of Cd^2+^ at a temporal resolution of 100 ms with our novel sensor. All our experiments were performed in tris buffer that mimics the artificial cerebellum fluid. We established a calibration curve resulting in a limit of detection (LOD) of 0.01 µM with a corresponding sensitivity of 418.02 nA/ µM. The sensor’s selectivity was evaluated in the presence of other metal ions, and it was noteworthy to observe that the sensor retained its ability to produce the distinctive Cd^2+^ CV, even when the concentration of other metal ions was 200 times higher than that of Cd^2+^. We also found that our sensor could detect free Cd^2+^ ions in the presence of complexing agents. Furthermore, we analyzed the solution chemistry of each of those Cd^2+^–ligand solutions using a geochemical model, PHREEQC. The concentrations of free Cd^2+^ ions determined through our electrochemical data align well with geochemical modeling data, thus validating the response of our novel sensor. Furthermore, we reassessed our sensor’s LOD in tris buffer based on the concentration of free Cd^2+^ ions determined through PHREEQC analysis, revealing an LOD of 0.00132 µM. We also demonstrated the capability of our sensor to detect Cd^2+^ ions in artificial urine samples, showcasing its potential for application in actual biological samples. To the best of our knowledge, this is the first AuNP-modified, CFM-based Cd^2+^ sensor capable of detecting ultra-low concentrations of free Cd^2+^ ions in different complex matrices, including artificial urine at a temporal resolution of 100 ms, making it an excellent analytical tool for future real-time, in vivo detection, particularly in the brain.

## 1. Introduction

The growing health concern of toxic heavy metal contamination has gained considerable attention worldwide. Among these metals, cadmium is ranked seventh on the priority list of hazardous substances on the Agency for Toxic Substances and Disease Registry [1]. Anthropogenic sources of environmental cadmium contamination [2] mainly stem from industrial pollutants such as electronic parts, paints, electroplating waste, and batteries [3,4]. Once cadmium enters the human body, it tends to accumulate and be stored in soft tissues [5] because of its long biological half-life of ~20–30 years and low excretion rate, it tends to accumulate and be stored in soft tissues [5], causing hazardous health issues such as nephrotoxicity, hepatotoxicity, endocrine and reproductive toxicities, and cancers leading to organ failure [6,7,8]. Furthermore, cadmium has also been linked to several neurodegenerative diseases, such as Alzheimer’s and Parkinson’s, due to its ability to permeate the blood-brain barrierblood–brain barrier and induce neurotoxicity [9,10]. It is crucial to develop an appropriate detection tool capable of detecting ultra-low concentrations of cadmium in both environmental and biological samples to alleviate the consequences of cadmium poisoning. 

Conventional methods of cadmium detection include inductively coupled plasma-mass spectroscopy [11], inductively coupled plasma–optical emission spectrometry [12], atomic fluorescence spectroscopy [13], and atomic absorption spectroscopy [14]. Despite the high sensitivity and low limit of detection (LOD) achieved with these non-electrochemical analysis methods, these tools have been used primarily with non-biological or post-mortem tissue samples, owing to their inherent limitations with real-time, in vivo measurements [15]. Such challenges include lengthy sample preparation that alters metal speciation, a critical factor determining metal toxicity, requiring expert personnel, sophisticated equipment, and lengthy analysis time. In contrast, electrochemical analysis methods provide rapid, easy, highly sensitive, and selective on-site measurements of metal speciation. Interestingly, Liu et al. have created a portable electrochemical system using graphene/ionic liquid-modified screen-printed electrodes to detect cadmium in soil samples via square-wave anodic stripping voltammetry [16]. Similarly, Singh and colleagues have developed a bioconjugated reduced graphene oxide-based nanocomposite for the electrochemical detection of cadmium in water using differential pulse voltammetry [17]. Furthermore, the Pathirathna group have reported a nanopipette capable of detecting cadmium in water samples via ion transfer at the interface between two immiscible electrolyte solutions [18]. While these in vitro studies exhibit promising results with non-biological samples, there is a notable gap in the literature concerning studies on a sensor capable of detecting ultra-low concentrations of cadmium ions in biological samples with a suitablee high temporal resolution for in vivo measurements.

In contrast to traditional electrochemical techniques, fast-scan cyclic voltammetry (FSCV) has emerged as a powerful electrochemical tool to detect neurotransmitters and toxic metal ions with high temporal resolution (100 ms). The Hashemi group have pioneered the development of the first FSCV-based metal sensor that can detect Cu^2+^ in aqueous solutions using carbon carbon-fiber microelectrodes (CFM) [19]. Similarly, the Hashemi group have reported a FSCV-based Pb^2+^ sensor [20]. Using a series of studies [21,22,23], the Hashemi group explored the fundamental mechanism of their metal sensors and developed mathematical relationships to predict the absolute concentrations of copper ions in complex matrices. Although these studies show that CFMs and FSCV are ideal candidates for developing metal sensors, the reported LODs are inadequate for detecting ultra-low concentrations in biological samples. Therefore, surface-modification strategies are needed to enhance the analyte adsorption on CFM surfaces and, thus, to improve the sensitivity of the sensor. Interestingly, Manring et al. [24] modified the bare CFM surfaces by electrodepositing polydopamine on the carbon surface to enhance the sensitivity of copper detection significantly. Similarly, Zestos and colleagues report using gold nanoparticles (AuNP)-modified CFMs for enhanced neurochemical detection via FSCV [25]. Moreover, AuNP has been used with other electrode materials and electrochemical techniques to detect toxic metal ions such as mercury [26]. 

In this study, we report using AuNP-modified CFMs for the electrochemical detection of Cd^2+^ via FSCV. We first optimized the electrochemical parameters for Cd^2+^ detection by systematically changing the potential window and scan rate via a series of experiments. We then constructed a calibration curve using the optimized electrochemical parameters to determine the linear range and LOD. We also tested the selectivity of our sensor in the presence of other metal ions. Our complexation studies show that our sensor is capable of detecting free cadmium ions in the presence of different ligands. In-depth complexation investigations using PHREEQC geochemical modeling enabled us to determine the concentration of free Cd^2+^ in the presence of various ligands, thus validating our FSCV data. Furthermore, we investigated our sensor’s performance in artificial urine spiked with various concentrations of Cd^2+^ and constructed a calibration curve to determine analytical parameters. To the best of our knowledge, this is the first study reporting the use of AuNP-modified CFMs to detect Cd^2+^ with high selectivity and sensitivity via FSCV. The ability of our sensor to detect ultra-low concentrations of Cd^2+^ in both artificial urine and a tris buffer that mimics artificial cerebellum fluid showcases the great potential of our sensor for future in vivo studies. 

## 2. Materials and Methods

### 2.1. Solutions

Unless otherwise specified, all chemicals were purchased from Sigma-Aldrich (St. Louis, MO, USA). Cadmium chloride (Alfa Aesar, Ward Hill, MA, USA) was used as the Cd^2+^ source. Cd^2+^ solutions were prepared in tris buffer composed of tris hydrochloride (15 mM), NaCl (140 mM), KCl (3.25 mM), CaCl_2_ (1.2 mM), NaH_2_PO_4_ (1.25 mM), MgCl_2_ (1.2 mM), and Na_2_SO_4_ (2.0 mM) at pH 7.4. 1% AuCl_3_ was used for gold nanoparticle electrodeposition (Salt Lake Metals, Nephi, UT, USA). Ethylenediaminetetraacetic acid (EDTA), dimercaptosuccinic acid (DMSA), diethylenetriaminepentaacetic acid (DTPA), and nitrilotriacetic acid (NTA) were used as model ligands to prepare Cd^2+^–ligand samples by mixing Cd^2+^ and ligand in a 1:1 ratio in tris buffer. CuSO_4_·5H_2_O, PbCl_2_, Co(OOCCH_3_)_2_·4H_2_O, and Mn(NO_3_)_2_·xH_2_O were used as the sources for the selectivity test in tris buffer. Artificial Urine Control (Innovating Science, Avon, NY, USA) mixed with 0.1 M KCl was used for urine measurements. 

### 2.2. Preparation of AuNP-Modified CFMs

Bare CFMs were constructed by utilizing electrostatic forces between a wire and carbon fibers (diameter: 7 µm, GoodFellow, Pittsburgh, PA, USA) to insert a single carbon fiber into borosilicate glass capillaries (internal diameter: 0.58 mm, external diameter: 1.0 mm, Sutter Instruments, Novato, CA, USA). The fiber-filled capillaries were pulled under gravity using a vertical micropipetmicropipette puller, PE-100 (Narishige Group, Setagaya-Ku, Tokyo, Japan), producing a carbon–glass seal. The pulled CFMs were then trimmed manually to 130–140 µm under an optical microscope. Electrochemical connection is was made with by inserting a connecting Ag wire and Hg. 

**Figure 1:**
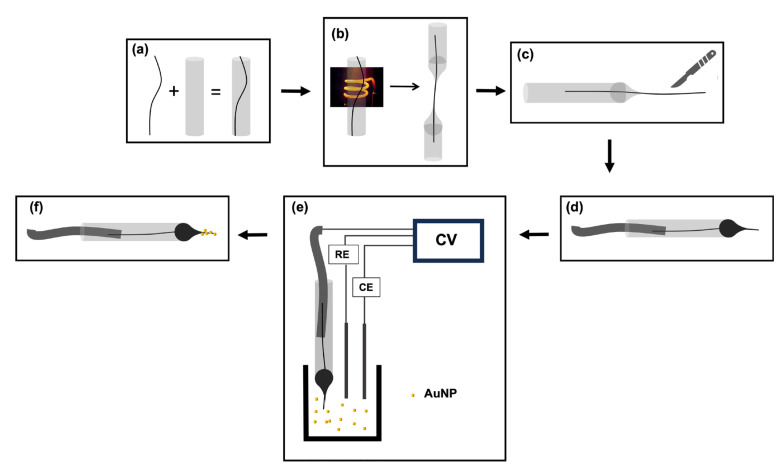
Schematic representation of fabrication of AuNP-modified CFMs. (**a**) Insertion of carbon fiber into a glass capillary. (**b**) Pulling fiber-filled capillaries into two halves under gravity using a heating coil in a vertical puller. (**c**) Trimming the exposed length of the carbon fiber manually using a scalpel blade under a microscope. (**d**) Making an electrochemical connection with Hg and an Ag wire. (**e**) Electrodeposition of AuNP via cyclic voltammetry (CV) using a three-electrode system using an in-house built Ag/AgCl electrode as the reference electrode (RE) and a Pt wire as the counter electrode (CE). (**f**) AuNP-modified CFM.

Consistent with Zestos’s electrodeposition of AuNPs [25], the surfaces of CFMs were modified by electrodepositing gold nanoparticles using 0.5 mM HAuCl_4_ mixed in 0.1 M KCl and cycling the potential from +0.2 V to −1.0 V at 50 mV/s for 10 cycles using a CHI660E potentiostat (CH Instruments, TX, USA) in a three-electrode system using a in-house built Ag/AgCl electrode as the reference electrode and a Pt wire (Alfa Aesar, MA, USA) as the counter electrode (Figure 1).

### 2.3. Electrochemical Measurements

All electrochemical measurements were performed in a two-electrode system with surface-modified CFMs as working electrodes and an in-house built Ag/AgCl electrode as the reference electrode. Data collection, data analysis, and background subtraction was were performed with by using a Quad-UEI system (Electronics Design Facility, University of North Carolina, Chapel Hill, NC, USA). Each electrochemical experiment was conducted with at least 3 CFMs four times (at least 12 runs in total). 

### 2.4. Solution Chemistry Analysis

The sSolution chemistry of Cd^2+^ in different matrices was analyzed using PHREEQC, a geochemical modeling software capable of determining speciation based on thermodynamic equilibrium. Stability constants during modeling were based on the NIST standard reference 46 database [27] in conjunction with other relevant literature [28], while additional constants for complexation with solutions were modeled in equilibrium with CO_2_(g) and O_2_(g) at 25 °C and 1 atm. 

## 3. Results and Discussion

### 3.1. FSCV of Cd^2+^ on Surface-Modified CFM

Bare CFMs have been widely employed with FSCV to detect a range of neurotransmitters [29,30,31], and toxic metal ions [19,20], attributing attributed to their advantageous characteristics, including hemispherical mass transport, low ohmic drop due to their smaller size, and high surface-adsorption ability resulting from surface oxygenated groups [31], resulting in higher sensitivity compared to other electrodes [29]. While FSCV offers many benefits, its adsorption-driven nature, as opposed to the diffusion-driven traditional slow-scan cyclic voltammetry, necessitates the optimization of electrochemical parameters for generating unique analyte-specific CVs when employing FSCV for the first time with a new analyte. Therefore, as pioneers in using FSCV to detect Cd^2+^ ions, we started our experiments by optimizing a suitable potential window and a scan rate to generate a distinctive CV for Cd^2+^ in tris buffer. We first used bare -CFMs and systematically changed the positive, negative, and resting potentials and scan rates (data not shown). However, despite our efforts, we did not observe any reproducible CV with redox peaks using bare CFMs. We then explored possible surface-modification strategies to improve surface adsorption of Cd^2+^ on CFM surfaces. Drawing inspiration from our previous study with surface-modified CFMs with electrodeposited polydopamine to yield a significant improvement in Cu^2+^ detection [24], we employed the same surface-modification protocol toward Cd^2+^ detection. However, we did not observe any promising CVs with polydopamine-deposited CFMs. 

**Figure 2:**
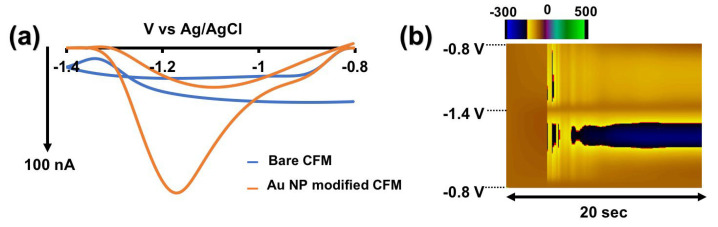
(**a**) Representative CVs obtained for 0.25 µM Cd^2+^ with AuNP-modified CFMs (orange CV) and bare CFMs (blue CV) in tris buffer. (**b**) Representative color plot obtained for 0.25 µM Cd^2+^ in tris buffer with AuNP-modified CFM.

Among several other surface modification strategies, we sought to modify our CFMs with AuNP following a simple approach reported by the Zestos group [25]. High conductivity, favorable catalytic properties, and the ability to prevent bio-fouling make AuNP a preferred choice for many electrochemical sensor modifications [25,26]. Interestingly, upon electrodepositing AuNP on bare CFMs, a unique Cd^2+^-specific CV appeared upon changing positive, negative, and resting potentials (Figure 2). After testing several combinations of positive, negative, and resting potentials, we observed the highest reduction current (at ~1.1 V) when scanning from −0.8 V to −1.4 V with a resting potential of −0.8 V. We attribute this distinctive peak to the reduction of Cd^2+^ to Cd^0^. However, no apparent oxidation peak was observed under any of the tested conditions. While a separate project is underway to comprehend the mechanism behind the sensitivity of AuNP-modified CFMs, this lack of oxidation peak could be attributed to the sluggish kinetics of Cd^0^ to Cd^2+^ oxidation under the faster scan rates employed in FSCV or a stable complex formed between AuNP and cadmium after the reduction of Cd^2+^ to Cd^0^. Moreover, in the FSCV analysis of most analytes, only the forward peak is was employed for both qualitative and quantitative purposes. For instance, dopamine--FSCV and serotonin--FSCV rely solely on the prominent forward oxidation peaks of dopamine [29] and serotonin CVs [32], whereas Cu-FSCV utilizes only the reduction peak that appears on the forward peak for analysis [19]. Thus, having only one peak is sufficient for further analysis.

**Figure 3:**
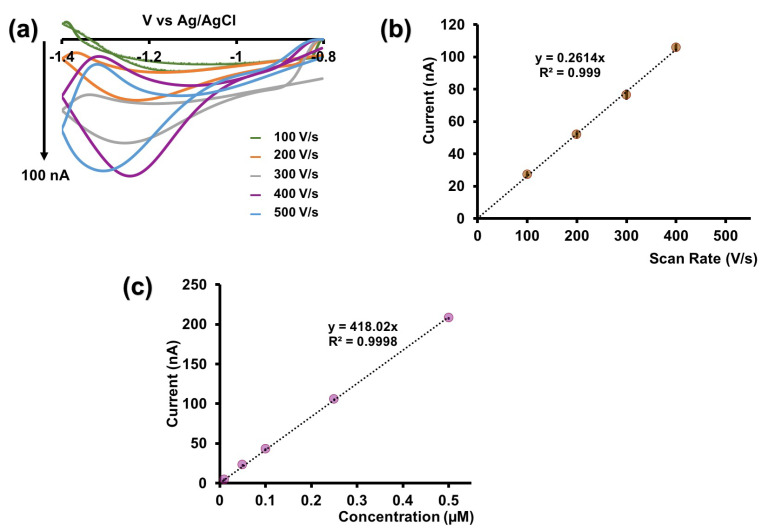
(**a**) Representative CVs obtained for 0.25 µM Cd^2+^ for each scan rate when potential was varied from −0.8 V to −1.4 V and back to −0.8 V. (**b**) Plot of maximum reduction peak current vs. scan rate. (**c**) Calibration curve for Cd^2+^ using AuNP-modified CFMs in tris buffer. The potential was cycled from −0.8 V to −1.4V at 400 V/s. Each data point represents the average oxidation current ± standard error of mean obtained for three CFMs with at least 4 replicate measurements for each CFM (minimum of 12 total replicates).

After optimizing the potential window and resting potential, we proceeded to optimize the scan rate. As depicted in Figure S1, the reduction current increases with the scan rate up to 400 V/s, reaching a plateau at 500 V/s. Although the current at 500 V/s is nearly identical to that at 400 V/s, the CV becomes broader and distorted at 500 V/s [19] (Figure 3a). Consequently, we selected 400 V/s as the optimum scan rate. Additionally, the R^2^ value of the linear range is approximately 0.99 (Figure 3b), indicative of an adsorption-driven response similar to that the previously reported for Cu-FSCV [19]. Using the optimized waveform (−0.8 V to −1.4 V at 400 V/s), we constructed a calibration curve for Cd^2+^ in tris buffer (Figure 3c and Figure S2) to establish analytical parameters. The linear range of our calibration curve extended up to 0.5 μM, with a LOD of 0.01 μM and a sensitivity of 418.02 nA/μM for our sensor. We evaluated the stability of our sensor by consecutively injecting Cd^2+^, and, as illustrated in Figure S3, it demonstrated excellent stability even after the surface modification with AuNPs.

### 3.2. Selectivity of Cd^2+^ Waveform

**Figure 4:**
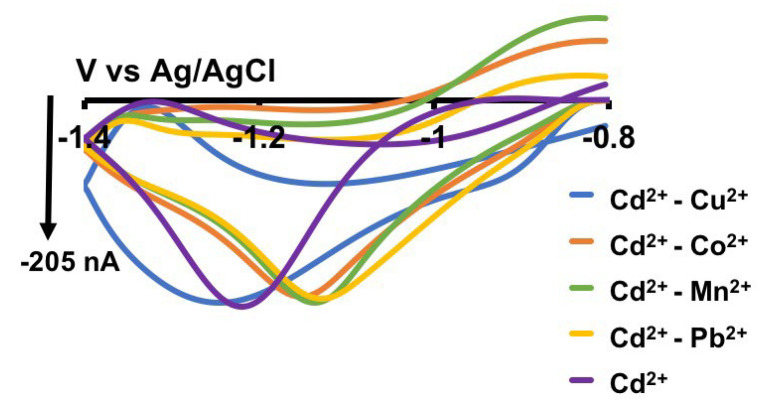
Representative CVs obtained for 0.5 µM Cd^2+^ in the presence 100 µM Cu^2+^ (blue CV), Co^2+^ (orange CV), Mn^2+^ (green CV), Pb^2+^ (yellow CV), and without (purple CV) in tris buffer.

It is crucial to assess the selectivity of any metal sensor against potential interfering metal ions for to obtaining accurate and robust qualitative and quantitative information. Therefore, we evaluated the selectivity of our Cd^2+^ sensor against Cu^2+^, Co^2+^, Mn^2+^, and Pb^2+^ by maintaining a concentration ratio of 1:200 for Cd^2+^ (0.5 µM) to other metal ions (100 µM). As illustrated in Figure 4, our sensor was able to generate a unique Cd^2+^ CV even in the presence of high concentrations of other metal ions, showcasing excellent selectivity. Minor shifts in peaks are expected as the matrix composition changes, and the observed shifts in some CVs in Figure 4 are considered normal. Additionally, as our tris buffer already contains high concentrations of Ca^2+^, Mg^2+^, and Na^+^, we did not perform additional selectivity tests with those ions.

### 3.3. Complexation Study 

When metal ions are present in the environment or the body, they primarily exist in complex bound forms [33]. Therefore, it is important to study the sensor’s ability to detect free Cd^2+^ ions, even in the presence of strong ligands that can form robust Cd^2+^–ligand complexes. To address this, we conducted a comprehensive complexation study using both electrochemical and geochemical modeling strategies. We utilized four model ligands, EDTA, DTPA, DMSA, and NTA, as complexing ligands [34].

**Figure 5:**
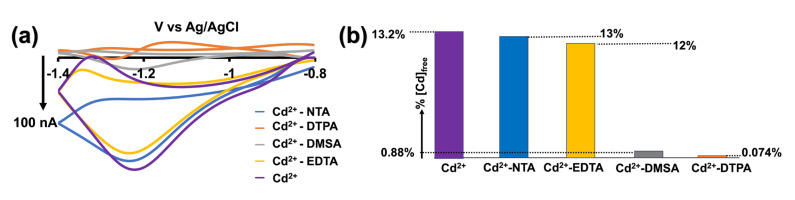
(**a**) Representative CVs obtained for 0.5 µM Cd^2+^ with the addition of 0.5 µM NTA (blue CV), DTPA (orange CV), DMSA (grey CV), EDTA (yellow CV), and without (purple CV) in tris buffer. (**b**) Bar graph showing the % [Cd]free in tris buffer (purple bar), with NTA (blue bar), EDTA (yellow bar), DMSA (grey bar), and DTPA (orange bar) acquired from PHREEQC geochemical modeling.

In this study, Cd^2+^ was injected into our tris buffer, and each ligand was subsequently injected one at a time, allowing sufficient time for forming of Cd^2+^–ligand complexes before extracting FSCV files. The rationale behind this experiment was that as we add ligands, they will form complexes with free Cd^2+^ ions, thereby reducing the concentration of free Cd^2+^ ions in the solution and resulting in a lower reduction current in the CVs. As shown in Figure 5a, a slight decrease in the reduction currents was observed upon the addition of NTA and EDTA, while the current drastically reduced upon adding DMSA and DTPA. Despite these changes, our sensor retained the unique shape of the Cd^2+^ CV, providing strong evidence of its capability to detect free Cd^2+^ even in a complex matrix. 

Although our experimental data aligned with our hypothesis, we sought to further validate this observation through an in-depth characterization of Cd^2+^ speciation in tris buffer using PHREEQC geochemical modeling. In this analysis, we utilized NIST standard reference database 46 and previously reported stability constants for Cd^2+^–ligands [27,28]. The results of our geochemical modeling revealed that only 13.2% of Cd^2+^ exists as free cadmium in tris buffer. In the presence of NTA, EDTA, DMSA, and DTPA, these values dropped to 13%, 12%, 0.88%, and 0.074% of free Cd^2+^ available with each ligand, respectively (Figure 5b). Notably, these values closely coincide with our electrochemical data, thereby validating our sensor’s ability to detect free cadmium with high accuracy when present with complex-forming ligands.

Considering the free Cd^2+^ found in the tris buffer, we re-evaluated our LOD obtained from Figure 3c to be enhanced to 0.00132 µM (0.148 ppb), establishing our sensor as the most sensitive FSCV -metal sensor reported to date [10,19,24]. Interestingly, as seen in Table 1, the LOD of our sensor is comparable with most of the previously reported voltammetric sensors. Moreover, this low LOD falls below the physiological toxic levels of Cd^2+^, making it an ideal sensor for detecting Cd^2+^ in biological samples.

### 3.4. Urine Analysis 

After entering the human body, cadmium is eliminated primarily gets eliminated through urine [6]. Given our ultimate goal of utilizing the sensor in both biological samples and in vivo applications, we conducted tests in artificial urine. Initially, we tested store-bought urine spiked with 0.25 µM Cd^2+^. However, the reduction peak appeared on the backward scan, indicating insufficient conductivity for Cd^2+^ reduction to Cd^0^ during the forward scan (Figure S4).

**Figure 6:**
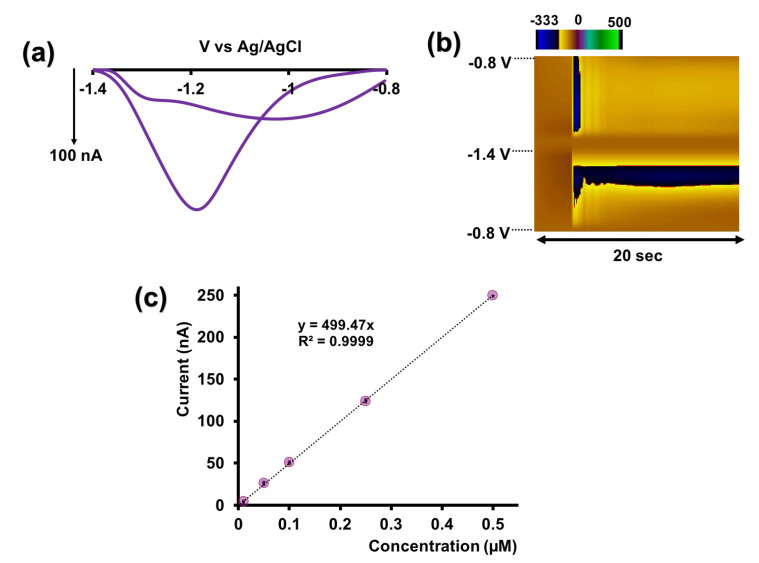
Representative CV (**a**) and color plot (**b**) obtained for 0.25 µM Cd^2+^ in artificial urine with 0.1 M KCl using AuNP-modified CFMs. (**c**) Calibration curve for Cd^2+^ using AuNP-modified CFMs in artificial urine with 0.1 M KCl. The potential was cycled from −0.8 V to −1.4 V at 400 V/s. Each data point represents the average oxidation current ± standard error of mean obtained for three CFMs with at least 4 replicate measurements for each CFM (minimum of 12 total replicates).

To address this, we added 0.1 M KCl to enhance conductivity and performed FSCV analysis. Excitingly, in Figure 6a,b, we successfully obtained Cd^2+^-CV and a color plot in the urine. Although the CV showed slight distortion compared to tris buffer, the characteristic peak remained identifiable (Figure 6a). Constructing a calibration curve (Figure 6c and Figure S5) resulted in a similar linear range and LOD as in tris buffer. The corresponding sensitivity in artificial urine was slightly higher (499.47 nA/µM), potentially due to differences in matrix components compared to tris buffer. Our sensor’s consistent and accurate detection of Cd^2+^ in both artificial urine and tris buffer, which mimics artificial cerebellum fluid, underscores its promising potential for future biological samples and in vivo studies.

## 4. Conclusions

The widespread prevalence and extreme toxicity of Cd^2+^ emphasize the urgent need for a sensor capable of rapid and precise detection of ultra-low cadmium levels. Conventional Cd^2+^ detection methods often modify metal speciation through sample pretreatment and are typically performed in vitro. While electrochemical methods have successfully detected Cd^2+^ in environmental samples, they are unsuitable for biological samples. Moreover, these methods cannot capture trace Cd^2+^ levels in the body and lack the necessary high temporal resolution for in vivo measurements.

In this study, we introduce the use of FSCV coupled with AuNP-modified CFMs for the electrochemical detection of Cd^2+^. AuNPs were electrodeposited onto CFM surfaces using a previously reported CV-based method. Detection parameters were optimized by adjusting the potential window and scan rate, demonstrating the waveform’s specificity for Cd^2+^ even in the presence of potential interfering toxic metals. Additionally, a thorough complexation study evaluated our sensor’s ability to detect free Cd^2+^ in the presence of model ligands. We validated our electrochemical data through comprehensive speciation analysis using PHREEQC, a widely used geochemical modeling platform.

Excitingly, our sensor successfully detected nanomolar concentrations of free Cd^2+^ in tris buffer and in the presence of ligands. Furthermore, we assessed our sensor’s stability in a new matrix, artificial urine, demonstrating excellent stability and sensitivity. This highlights our sensor’s potential for future biological samples and in vivo measurements. To our knowledge, this study represents the first application of FSCV coupled with surface-modified CFMs for the electrochemical detection of Cd^2+^, showcasing the highest sensitivity among all reported FSCV metal sensors.

## Figures and Tables

**Table 1:** Comparison of previously reported Cd sensors related to our research.

Electrochemical Method	Sensor	LOD (ppb)	Reference
Electrochemical Impedance Spectroscopy	AuNPs/CNTS/cChitosan nanocomposite aptamer	2.2 × 10^−6^	[35]
Anodic Stripping Voltammetry	Ion-printed polymer on glassy carbon electrode	7.9 × 10^−6^	[36]
Amperometry	Pt/PANI-co-PDTDA/HRP biosensor	0.0008	[37]
Adsorptive Stripping Voltammetry	Sulfisoxazole nanofilm on glassy carbon electrode	0.003	[38]
Square Square-Wave Anodic Stripping Voltammetry	Glassy carbon electrode modified with poly(1,8-diaminonaphthalene) coated with silver nanoparticles	0.019	[39]
Cyclic Voltammetry and Electrochemical Impedance Spectroscopy	Screen Screen-printed gold electrode with amino-modified aptamer	0.03	[40]
Cyclic Voltammetry	Enzymic membrane with screen screen-printed electrodes biosensor	0.05	[41]
Anodic Stripping Voltammetry	Bismuth bBulk eElectrode	0.054	[42]
Square-Wave Anodic Stripping Voltammetry	Co_3_O_4_ nanocrystals/rGO	0.062	[43]
Anodic Stripping Voltammetry	Glassy carbon electrode modified with nafion-CSB and in-situin situ mercury film	0.08	[44]
Amperometry	Enzyme inhibition biosensor based on indium tin oxide nanoparticles, hexaammineruthenium (III) chloride, and chitosan-modified glassy carbon electrode	0.11	[45]
Differential Pulse Voltammetry	Hexagonal mMesoporous sSilica-iImmobilized qQuercetin-m Modified cCarbon pPaste eElectrode	0.11	[46]
Square Square-Wave Anodic Stripping Voltammetry	Electropolymerized ion-imprinted poly PoPD/electrochemical reduced graphene oxide composite on glassy carbon electrode	0.13	[47]
Differential Pulse Anodic Stripping Voltammetry	Glassy carbon electrode modified with g-C_3_N_4_ and SnO_2_ NPs	0.16	[48]
Differential Pulse Anodic Stripping Voltammetry	Glassy carbon electrode modified with rGO and in-situin situ mercury film	0.17	[49]
Cloud Cloud-Point Extraction Anodic Stripping Voltammetry	Mercury-coated glassy carbon electrode	0.2	[50]
Differential Pulse Anodic Stripping Voltammetry	Glassy carbon electrode modified with BiSn alloy nanoparticles and nafion	0.33	[51]
Anodic Stripping Voltammetry	Glassy carbon electrode modified with poly(4-azulen-1-yl-2,6-bis(2-thienyl)pyridine) complexing films	1.12	[52]
Square Square-Wave Anodic Stripping Voltammetry	Pd at PAC-modified glassy carbon electrode	1.5	[53]
Square Square-Wave Stripping Voltammetry	Glassy carbon electrode modified with magnetite nanoparticles and fluorinated multiwalled carbon nanotubes	1.57	[54]
Square Square-Wave Anodic Stripping Voltammetry	3D-printed electrode fabricated using a conductive composite of polylactic acid containing carbon black	2.9	[55]

## Data Availability

All the necessary data are available in the article.

## Supplementary Materials

The following supporting information can be downloaded at: https://www.mdpi.com/article/10.3390/mi15030294/s1.
