# Supplementary material for: Gold Nanoparticle-Modified Carbon-Fiber Microelectrodes for the Electrochemical Detection of Cd2+ via Fast-Scan Cyclic Voltammetry
Source: Micromachines (Basel). 2024 Feb 21;15(3):294. doi: 10.3390/mi15030294 (PMC10971841; doi:10.3390/mi15030294)
Supplement: Supplementary file 1 [file micromachines-15-00294-s001.zip › micromachines-2861954-supplementary.pdf]

Supplementary Material:

Gold Nanoparticle-Modified Carbon-Fiber Microelectrodes for the Electrochemical Detection of Cd^{2+} via Fast-Scan Cyclic Voltammetry

Noel Manring, Miriam Strini, Gene Koifman, Jessica L. Smeltz and Pavithra Pathirathna *

Department of Chemistry and Chemical Engineering, Florida Institute of Technology, 150 W. University Blvd, Melbourne, FL 32901, USA; nmanring2020@my.fit.edu (N.M.); mstrini2021@my.fit.edu (M.S.); gkoifman2021@my.fit.edu (G.K.); jsmeltz@fit.edu (J.L.S.)
* Correspondence: ppathirathna@fit.edu

Table of Contents:

1. Optimization of scan rate
2. Calibration curve of Cd^{2+} in tris buffer
3. Stability test
4. Detection of Cd^{2+} in artificial urine
5. Calibration curve of Cd^{2+} in artificial urine
6. PHREEQC output file for Cd^{2+} in tris
7. PHREEQC output file for Cd^{2+}+NTA in tris
8. PHREEQC output file for Cd^{2+}+EDTA in tris
9. PHREEQC output file for Cd^{2+}+DMSA in tris
10. PHREEQC output file for Cd^{2+}+DPTA in tris

Optimization of scan rate: After optimizing positive, negative, and resting potentials, we explored various scan rates from 100 V/s to 500 V/s to identify the optimal scan rate for generating the maximum reduction current and a non-distorted Cd^{2+}-CV. As depicted in Figure S1, the reduction current for Cd^{2+} increased up to 400 V/s, plateauing at 500 V/s. While the current at 500 V/s was similar to that at 400 V/s, the CV shape appeared distorted. Consequently, 400 V/s was selected as the optimum scan rate.

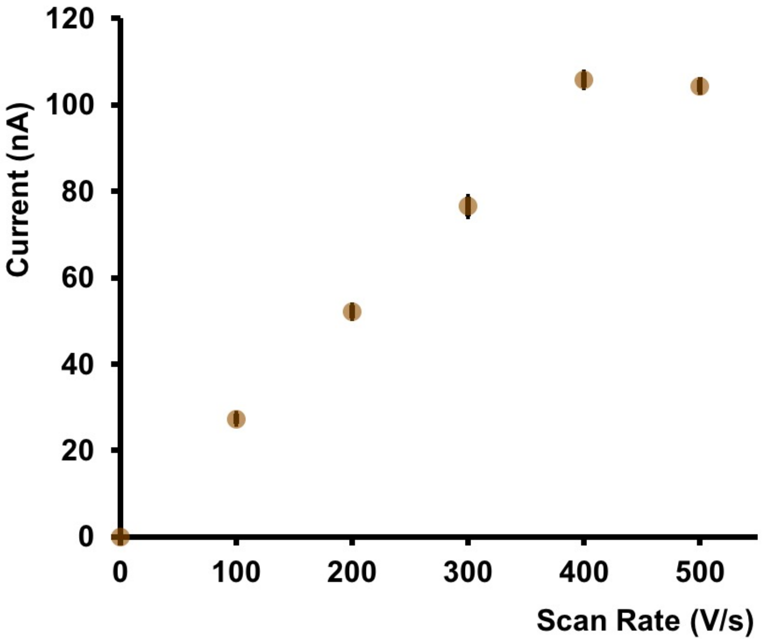

Figure S1. The plot of maximum reduction current vs. scan rate for 0.25 μM Cd^{2+} in tris buffer upon cycling the potential was from -0.8 V to -1.4 V and back to -1.4 V. Each data point represents the average reduction current ± standard error of mean obtained for three CFMs with at least four replicate measurements for each CFM (minimum of 12 total replicates).

Calibration curve of Cd^{2+} in tris buffer: We varied the concentration of Cd^{2+} from 0.01 µM to 5 µM in tris buffer and measured the resulting maximum reduction current with our AuNP sensor, utilizing the optimized waveform. This allowed us to determine the limit of detection, linear range, and sensitivity of the sensor.

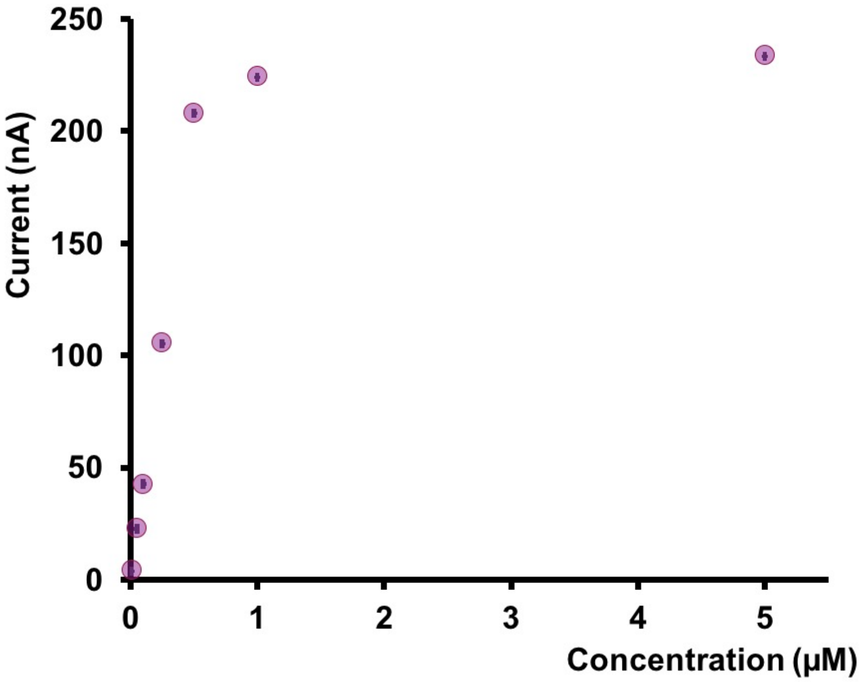

Figure S2. Complete calibration curve of Cd^{2+} in tris buffer with AuNP-modified CFMs. The potential was cycled from -0.8 V to -1.4 V at 400 V/s. Each data point represents the average reduction current ± standard error of mean obtained for three CFMs with at least 4 replicate measurements for each CFM (minimum of 12 total replicates).

Stability test: We assessed the sensitivity of our sensor by consecutively injecting Cd^{2+} into it. As depicted in Figure S3, after 30 consecutive injections of 0.25 µM Cd^{2+}, the maximum reduction current remained stable, indicating excellent stability of our sensor.

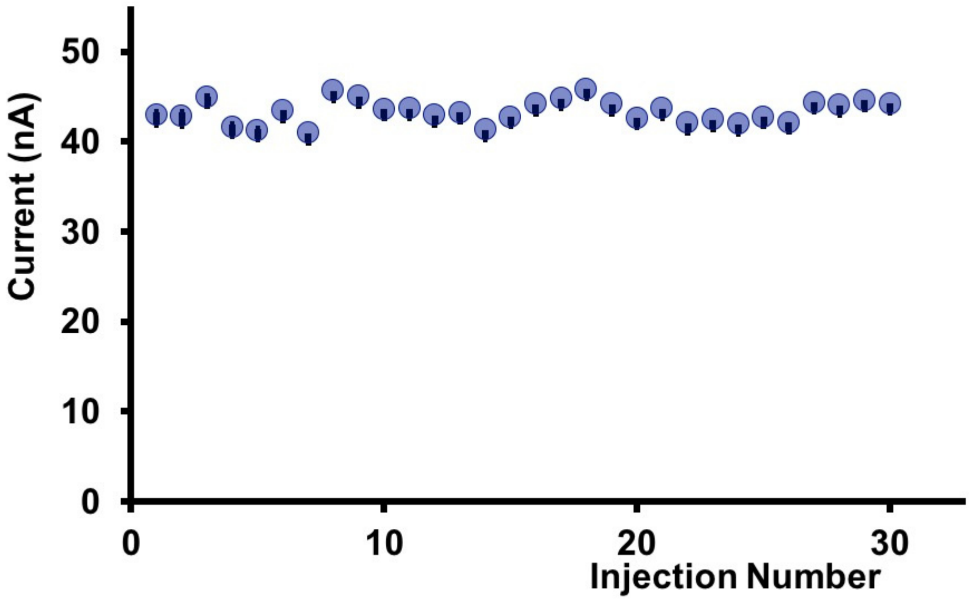

Figure S3. Maximum FSCV reduction current obtained with AuNP-modified CFMs upon successive injections of 0.25 µM Cd^{2+} onto CFMs in tris buffer. Each data point represents the average reduction current ± standard error of mean obtained for three CFMs with at least 4 replicate measurements for each CFM (minimum of 12 total replicates).

Detection of Cd^{2+} in artificial urine: We initially examined our sensor's reaction to spiked Cd^{2+} in store-bought artificial urine. As illustrated in Figure S4, we noted a reduction current for 0.25 µM Cd^{2+} on the backward scan, signifying insufficient conductivity for a reduction peak on the forward scan. Consequently, we supplemented the urine with 0.1 M KCl to enhance conductivity, allowing the generation of a typical Cd^{2+}-CV for subsequent analysis.

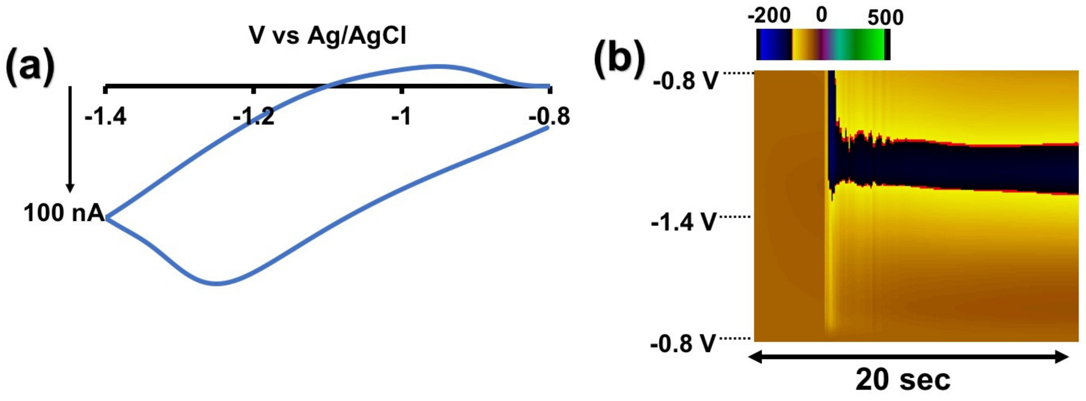

Figure S4. Representative CV (a) and color plot (b) for 0.25 µM Cd^{2+} in artificial control urine.

Calibration curve in artificial urine: Upon adding 0.1 M KCl to artificial urine, we systematically varied the concentration of spiked Cd^{2+} from 0.01 μM to 5 μM to establish a calibration curve. As depicted in Figure S5, the linear range extended up to 0.5 μM, with a plateau observed from 1-5 μM.

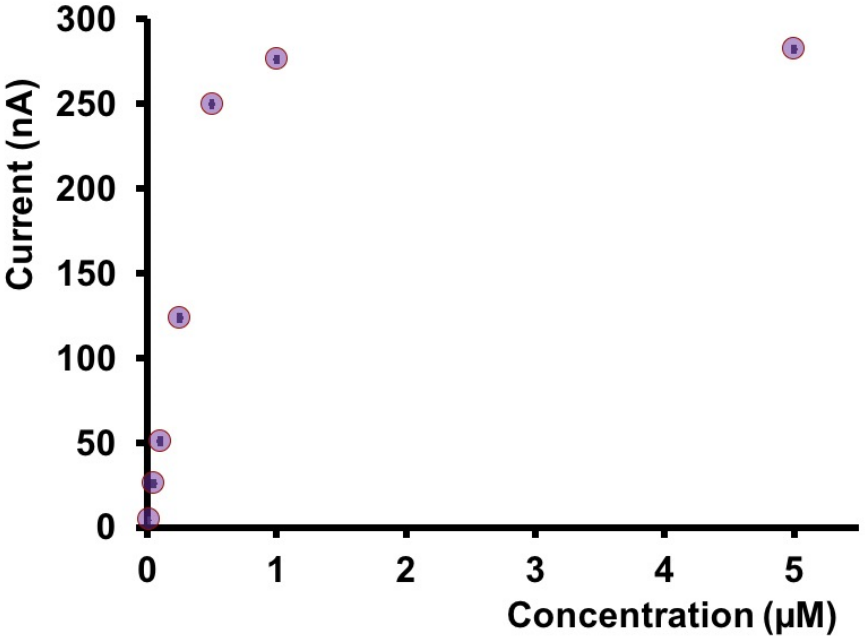

Figure S5. Complete calibration curve of Cd^{2+} in artificial urine with 0.1 M KCl with AuNP-modified CFMs. The potential was cycled from -0.8 V to -1.4 V at 400 V/s. Each data point represents the average reduction current ± standard error of mean obtained for three CFMs with at least 4 replicate measurements for each CFM (minimum of 12 total replicates).

PHREEQC output file for Cd^{2+} in tris

Input file: cd-tris
Output file: cd-tris.out
Database file: C:\Users\genek\Documents\Research\phreeqc-3.7.3-15968-x64\database\phreeqc.dat

```
------------------
Reading data base.
------------------

	SOLUTION_MASTER_SPECIES
	SOLUTION_SPECIES
	PHASES
	EXCHANGE_MASTER_SPECIES
	EXCHANGE_SPECIES
	SURFACE_MASTER_SPECIES
	SURFACE_SPECIES
	RATES
	END
------------------------------------
Reading input data for simulation 1.
------------------------------------

	SOLUTION 1 Test2
		temp 25.0
		pH 7.3
		pe 13.7848
		redox O(-2)/O(0)
		units mmol/L
		Ca 1.198
		K  3.26
		Na 243
		Mg 1.22
		Cl 163
		S(6) 2 P
		1.26
		O(0) 0.284
		C(4) 38.63
		Cd 0.0005
		Trizma 15
	END
-------------------------------------------
Beginning of initial solution calculations.
-------------------------------------------
Initial solution 1.		Test2

-----------------------------		Solution composition------------------------------

	Elements		Molality	Moles

	C(4)		3.926e-02	3.926e-02
	Ca		1.217e-03	1.217e-03
	Cd		5.081e-07	5.081e-07
	Cl		1.656e-01	1.656e-01
	K		3.313e-03	3.313e-03
	Mg		1.240e-03	1.240e-03
	Na		2.469e-01	2.469e-01
	O(0)		2.886e-04	2.886e-04
	P		1.280e-03	1.280e-03
	S(6)		2.032e-03	2.032e-03
	Trizma		1.524e-02	1.524e-02
```

----------------------------- Description of solution----------------------------

pH	=	7.300
pe	=	13.785
Specific Conductance (µS/cm, 25°C)	=	21158
Density (g/cm³)	=	1.00940
Volume (L)	=	1.00683
Activity of water	=	0.992
Ionic strength (mol/kgw)	=	2.403e-01
Mass of water (kg)	=	1.000e+00
Total alkalinity (eq/kg)	=	2.258e-02
Total CO2 (mol/kg)	=	3.926e-02
Temperature (°C)	=	25.00
Electrical balance (eq)	=	6.160e-02
Percent error, 100*(Cat-|An|)/(Cat+|An|)	=	13.06
Iterations	=	10
Total H	=	1.110656e+02
Total O	=	5.563486e+01

---------------------------------- Redox couples---------------------------------

Redox couple	pe	Eh (volts)
O(-2)/O(0)	13.2676	0.7849

----------------------------- Distribution of species----------------------------

Species	Molality	Activity	Log Molality	Log Activity	Log Gamma	mole V cm³/mol
OH-	2.895e-07	2.003e-07	-6.538	-6.698	-0.160	-3.41
H+	6.340e-08	5.012e-08	-7.198	-7.300	-0.102	0.00
H2O	5.551e+01	9.920e-01	1.744	-0.004	0.000	18.07
C(4)	3.926e-02					
HCO3-	3.374e-02	2.480e-02	-1.472	-1.606	-0.134	25.67
CO2	2.665e-03	2.817e-03	-2.574	-2.550	0.024	34.43
NaHCO3	2.379e-03	2.514e-03	-2.624	-2.600	0.024	1.80
MgHCO3+	1.366e-04	9.625e-05	-3.865	-4.017	-0.152	5.73
CaHCO3+	1.313e-04	9.800e-05	-3.882	-4.009	-0.127	9.89
NaCO3-	1.053e-04	7.790e-05	-3.977	-4.108	-0.131	0.53
CO3-2	7.953e-05	2.320e-05	-4.099	-4.634	-0.535	-3.43
CaCO3	1.142e-05	1.207e-05	-4.942	-4.918	0.024	-14.60
MgCO3	6.950e-06	7.345e-06	-5.158	-5.134	0.024	-17.09
(CO2)2	1.378e-07	1.457e-07	-6.861	-6.837	0.024	68.87
CdHCO3+	2.121e-08	1.569e-08	-7.673	-7.804	-0.131	(0)
CdCO3	3.488e-10	3.687e-10	-9.457	-9.433	0.024	(0)
Cd(CO3)2-2	9.042e-11	2.705e-11	-10.044	-10.568	-0.524	(0)
Ca	1.217e-03					
Ca+2	1.012e-03	3.098e-04	-2.995	-3.509	-0.514	-17.14
CaHCO3+	1.313e-04	9.800e-05	-3.882	-4.009	-0.127	9.89
CaHPO4	3.671e-05	3.880e-05	-4.435	-4.411	0.024	(0)
CaSO4	2.102e-05	2.222e-05	-4.677	-4.653	0.024	7.50
CaCO3	1.142e-05	1.207e-05	-4.942	-4.918	0.024	-14.60
CaPO4-	2.492e-06	1.832e-06	-5.603	-5.737	-0.134	(0)
CaH2PO4+	1.989e-06	1.462e-06	-5.701	-5.835	-0.134	(0)
CaOH+	1.376e-09	1.017e-09	-8.861	-8.992	-0.131	(0)
CaHSO4+	9.898e-12	7.320e-12	-11.004	-11.135	-0.131	(0)
Cd	5.081e-07					
CdCl+	3.003e-07	2.221e-07	-6.522	-6.653	-0.131	7.92
CdCl2	1.019e-07	1.077e-07	-6.992	-6.968	0.024	23.14
Cd+2	6.685e-08	2.000e-08	-7.175	-7.699	-0.524	-17.84

	CdHCO3+	2.121e-08	1.569e-08	-7.673	-7.804	-0.131	(0)
	CdCl3-	1.068e-08	7.898e-09	-7.971	-8.102	-0.131	69.75
	CdHPO4	2.728e-09	2.883e-09	-8.564	-8.540	0.024	(0)
	CdSO4	2.202e-09	2.327e-09	-8.657	-8.633	0.024	78.05
	CdOHCl	1.718e-09	1.816e-09	-8.765	-8.741	0.024	(0)
	CdCO3	3.488e-10	3.687e-10	-9.457	-9.433	0.024	(0)
	Cd(CO3)2-2	9.042e-11	2.705e-11	-10.044	-10.568	-0.524	(0)
	CdOH+	4.452e-11	3.293e-11	-10.351	-10.482	-0.131	(0)
	Cd(SO4)2-2	3.440e-11	1.029e-11	-10.463	-10.987	-0.524	-104.89
	CdH2PO4+	2.500e-11	1.849e-11	-10.602	-10.733	-0.131	(0)
	Cd(HPO4)2-2	1.749e-11	5.233e-12	-10.757	-11.281	-0.524	(0)
	Cd(OH)2	3.312e-14	3.500e-14	-13.480	-13.456	0.024	(0)
	Cd2OH+3	4.872e-17	3.226e-18	-16.312	-17.491	-1.179	(0)
	Cd(OH)3-	1.051e-19	7.773e-20	-18.978	-19.109	-0.131	(0)
	Cd(OH)4-2	4.582e-26	1.371e-26	-25.339	-25.863	-0.524	(0)
	Trizma2.2Cd+2	1.792e-39	5.361e-40	-38.747	-39.271	-0.524	(0)
Cl		1.656e-01					
	Cl-	1.656e-01	1.163e-01	-0.781	-0.935	-0.154	18.50
	CdCl+	3.003e-07	2.221e-07	-6.522	-6.653	-0.131	7.92
	CdCl2	1.019e-07	1.077e-07	-6.992	-6.968	0.024	23.14
	CdCl3-	1.068e-08	7.898e-09	-7.971	-8.102	-0.131	69.75
	CdOHCl	1.718e-09	1.816e-09	-8.765	-8.741	0.024	(0)
H(0)		0.000e+00					
	H2	0.000e+00	0.000e+00	-44.309	-44.285	0.024	28.61
K		3.313e-03					
	K+	3.303e-03	2.304e-03	-2.481	-2.637	-0.156	9.38
	KSO4-	8.887e-06	6.532e-06	-5.051	-5.185	-0.134	34.54
	KHPO4-	1.397e-06	1.027e-06	-5.855	-5.989	-0.134	38.82
Mg		1.240e-03					
	Mg+2	1.007e-03	3.317e-04	-2.997	-3.479	-0.482	-20.86
	MgHCO3+	1.366e-04	9.625e-05	-3.865	-4.017	-0.152	5.73
	MgHPO4	5.316e-05	5.618e-05	-4.274	-4.250	0.024	(0)
	MgSO4	2.968e-05	3.137e-05	-4.528	-4.504	0.024	5.84
	MgCO3	6.950e-06	7.345e-06	-5.158	-5.134	0.024	-17.09
	MgPO4-	3.600e-06	2.646e-06	-5.444	-5.577	-0.134	(0)
	MgH2PO4+	2.712e-06	1.993e-06	-5.567	-5.700	-0.134	(0)
	MgOH+	3.158e-08	2.384e-08	-7.501	-7.623	-0.122	(0)
Na(1)		2.469e-01					
	Na+	2.439e-01	1.803e-01	-0.613	-0.744	-0.131	-0.88
	NaHCO3	2.379e-03	2.514e-03	-2.624	-2.600	0.024	1.80
	NaSO4-	4.959e-04	3.645e-04	-3.305	-3.438	-0.134	17.97
	NaHPO4-	1.093e-04	8.031e-05	-3.961	-4.095	-0.134	50.71
	NaCO3-	1.053e-04	7.790e-05	-3.977	-4.108	-0.131	0.53
	NaOH	3.417e-18	3.612e-18	-17.466	-17.442	0.024	(0)
O(0)		2.886e-04					
	O2	1.443e-04	1.525e-04	-3.841	-3.817	0.024	30.40
P		1.280e-03					
	HPO4-2	8.182e-04	2.285e-04	-3.087	-3.641	-0.554	8.44
	H2PO4-	2.509e-04	1.844e-04	-3.600	-3.734	-0.134	33.91
	NaHPO4-	1.093e-04	8.031e-05	-3.961	-4.095	-0.134	50.71
	MgHPO4	5.316e-05	5.618e-05	-4.274	-4.250	0.024	(0)
	CaHPO4	3.671e-05	3.880e-05	-4.435	-4.411	0.024	(0)
	MgPO4-	3.600e-06	2.646e-06	-5.444	-5.577	-0.134	(0)
	MgH2PO4+	2.712e-06	1.993e-06	-5.567	-5.700	-0.134	(0)
	CaPO4-	2.492e-06	1.832e-06	-5.603	-5.737	-0.134	(0)
	CaH2PO4+	1.989e-06	1.462e-06	-5.701	-5.835	-0.134	(0)
	KHPO4-	1.397e-06	1.027e-06	-5.855	-5.989	-0.134	38.82
	PO4-3	4.802e-08	2.055e-09	-7.319	-8.687	-1.369	-19.58
	CdHPO4	2.728e-09	2.883e-09	-8.564	-8.540	0.024	(0)
	H3PO4	1.288e-09	1.361e-09	-8.890	-8.866	0.024	47.41
	CdH2PO4+	2.500e-11	1.849e-11	-10.602	-10.733	-0.131	(0)

Cd(HPO4)2-2		1.749e-11	5.233e-12	-10.757	-11.281	-0.524	(0)
S(6)	2.032e-03						
SO4-2		1.477e-03	4.034e-04	-2.831	-3.394	-0.564	16.24
NaSO4-		4.959e-04	3.645e-04	-3.305	-3.438	-0.134	17.97
MgSO4		2.968e-05	3.137e-05	-4.528	-4.504	0.024	5.84
CaSO4		2.102e-05	2.222e-05	-4.677	-4.653	0.024	7.50
KSO4-		8.887e-06	6.532e-06	-5.051	-5.185	-0.134	34.54
HSO4-		2.658e-09	1.966e-09	-8.575	-8.706	-0.131	40.69
CdSO4		2.202e-09	2.327e-09	-8.657	-8.633	0.024	78.05
Cd(SO4)2-2		3.440e-11	1.029e-11	-10.463	-10.987	-0.524	-104.89
CaHSO4+		9.898e-12	7.320e-12	-11.004	-11.135	-0.131	(0)
Trizma	1.524e-02						
TrizmaH+		1.524e-02	1.127e-02	-1.817	-1.948	-0.131	(0)
Trizma		1.391e-17	1.470e-17	-16.857	-16.833	0.024	(0)
Trizma2.2Cd+2		1.792e-39	5.361e-40	-38.747	-39.271	-0.524	(0)

------------------------------ Saturation indices------------------------------

Phase	SI**	log IAP	log K(298 K, 1 atm)	
Anhydrite	-2.63	-6.90	-4.28	CaSO4
Aragonite	0.19	-8.14	-8.34	CaCO3
Calcite	0.34	-8.14	-8.48	CaCO3
Cd(OH)2	-6.76	6.89	13.65	Cd(OH)2
Cd3(PO4)2	-7.87	-40.47	-32.60	Cd3(PO4)2
CdSO4	-10.99	-11.09	-0.10	CdSO4
CO2(g)	-1.08	-2.55	-1.47	CO2
Dolomite	0.83	-16.26	-17.08	CaMg(CO3)2
Gypsum	-2.33	-6.91	-4.58	CaSO4:2H2O
H2(g)	-41.18	-44.29	-3.10	H2
H2O(g)	-1.51	-0.00	1.50	H2O
Halite	-3.25	-1.68	1.57	NaCl
Hydroxyapatite	4.15	0.73	-3.42	Ca5(PO4)3OH
O2(g)	-0.92	-3.82	-2.89	O2
Otavite	-0.23	-12.33	-12.10	CdCO3
Sylvite	-4.47	-3.57	0.90	KCl

**For a gas, SI = log10(fugacity). Fugacity = pressure * phi / 1 atm.
For ideal gases, phi = 1.

End of simulation.

Reading input data for simulation 2.

End of Run after 10.399 Seconds.

PHREEQC output file for Cd^{2+}+NTA in tris

Input file: cd-tris-nta.txt
Output file: cd-tris-nta.txt.out
Database file: C:\Users\genek\Documents\Research\phreeqc-3.7.3-15968-x64\database\phreeqc.dat

----
Reading data base.

```
SOLUTION_MASTER_SPECIES
SOLUTION_SPECIES
PHASES
EXCHANGE_MASTER_SPECIES
EXCHANGE_SPECIES
SURFACE_MASTER_SPECIES
SURFACE_SPECIES
RATES
END
```

Reading input data for simulation 1.

```
SOLUTION 1 Test2
    temp 25.0
    pH 7.3
    pe 13.7848
    redox O(-2)/O(0)
    units mmol/L
    Ca 1.198
    K  3.26
    Na 243
    Mg 1.22
    Cl 163
    S(6) 2 P
    1.26
    O(0) 0.284
    C(4) 38.63
    Cd 0.0005
    Nta 0.0005
    Trizma 15
END
```

Beginning of initial solution calculations.

Initial solution 1. **Test2**

-----------------------------**Solution composition**-----------------------------

Elements	Molality	Moles
C(4)	3.926e-02	3.926e-02
Ca	1.217e-03	1.217e-03
Cd	5.081e-07	5.081e-07
Cl	1.656e-01	1.656e-01
K	3.313e-03	3.313e-03
Mg	1.240e-03	1.240e-03
Na	2.469e-01	2.469e-01
Nta	5.081e-07	5.081e-07
O(0)	2.886e-04	2.886e-04
P	1.280e-03	1.280e-03

S(6)	2.032e-03	2.032e-03

-1-

Trizma	1.524e-02	1.524e-02

----------------------------Description of solution----------------------------

pH	=	7.300
pe	=	13.785
Specific Conductance (µS/cm, 25°C)	=	21158
Density (g/cm³)	=	1.00940
Volume (L)	=	1.00683
Activity of water	=	0.992
Ionic strength (mol/kgw)	=	2.403e-01
Mass of water (kg)	=	1.000e+00
Total alkalinity (eq/kg)	=	2.258e-02
Total CO2 (mol/kg)	=	3.926e-02
Temperature (°C)	=	25.00
Electrical balance (eq)	=	6.160e-02
Percent error, 100*(Cat-|An|)/(Cat+|An|)	=	13.06
Iterations	=	10
Total H	=	1.110656e+02
Total O	=	5.563486e+01

---------------------------------Redox couples---------------------------------

Redox couple	pe	Eh (volts)
O(-2)/O(0)	13.2676	0.7849

----------------------------Distribution of species----------------------------

Species	Molality	Activity	Log Molality	Log Activity	Log Gamma	mole V cm³/mol
OH-	2.895e-07	2.003e-07	-6.538	-6.698	-0.160	-3.41
H+	6.340e-08	5.012e-08	-7.198	-7.300	-0.102	0.00
H2O	5.551e+01	9.920e-01	1.744	-0.004	0.000	18.07
C(4)	3.926e-02					
HCO3-	3.374e-02	2.480e-02	-1.472	-1.606	-0.134	25.67
CO2	2.665e-03	2.817e-03	-2.574	-2.550	0.024	34.43
NaHCO3	2.379e-03	2.514e-03	-2.624	-2.600	0.024	1.80
MgHCO3+	1.366e-04	9.625e-05	-3.865	-4.017	-0.152	5.73
CaHCO3+	1.313e-04	9.800e-05	-3.882	-4.009	-0.127	9.89
NaCO3-	1.053e-04	7.790e-05	-3.977	-4.108	-0.131	0.53
CO3-2	7.953e-05	2.320e-05	-4.099	-4.634	-0.535	-3.43
CaCO3	1.142e-05	1.207e-05	-4.942	-4.918	0.024	-14.60
MgCO3	6.950e-06	7.345e-06	-5.158	-5.134	0.024	-17.09
(CO2)2	1.378e-07	1.457e-07	-6.861	-6.837	0.024	68.87
CdHCO3+	2.092e-08	1.547e-08	-7.679	-7.810	-0.131	(0)
CdCO3	3.441e-10	3.636e-10	-9.463	-9.439	0.024	(0)
Cd(CO3)2-2	8.918e-11	2.668e-11	-10.050	-10.574	-0.524	(0)
Ca	1.217e-03					
Ca+2	1.012e-03	3.097e-04	-2.995	-3.509	-0.514	-17.14
CaHCO3+	1.313e-04	9.800e-05	-3.882	-4.009	-0.127	9.89
CaHPO4	3.671e-05	3.880e-05	-4.435	-4.411	0.024	(0)
CaSO4	2.102e-05	2.222e-05	-4.677	-4.653	0.024	7.50
CaCO3	1.142e-05	1.207e-05	-4.942	-4.918	0.024	-14.60
CaPO4-	2.492e-06	1.832e-06	-5.603	-5.737	-0.134	(0)
CaH2PO4+	1.989e-06	1.462e-06	-5.701	-5.835	-0.134	(0)
CaNta-	5.297e-08	3.918e-08	-7.276	-7.407	-0.131	(0)
CaOH+	1.376e-09	1.017e-09	-8.862	-8.993	-0.131	(0)
CaHSO4+	9.897e-12	7.320e-12	-11.004	-11.135	-0.131	(0)
Ca(Nta)2-4	5.479e-14	4.391e-16	-13.261	-15.357	-2.096	(0)

Cd	5.081e-07					
CdCl+	2.962e-07	2.191e-07	-6.528	-6.659	-0.131	7.92
CdCl2	1.005e-07	1.062e-07	-6.998	-6.974	0.024	23.14
Cd+2	6.593e-08	1.973e-08	-7.181	-7.705	-0.524	-17.84
CdHCO3+	2.092e-08	1.547e-08	-7.679	-7.810	-0.131	(0)
CdCl3-	1.053e-08	7.790e-09	-7.977	-8.108	-0.131	69.75
CdNta-	6.968e-09	5.154e-09	-8.157	-8.288	-0.131	(0)
CdHPO4	2.691e-09	2.844e-09	-8.570	-8.546	0.024	(0)
CdSO4	2.172e-09	2.295e-09	-8.663	-8.639	0.024	78.05
CdOHCl	1.694e-09	1.791e-09	-8.771	-8.747	0.024	(0)
CdCO3	3.441e-10	3.636e-10	-9.463	-9.439	0.024	(0)
Cd(CO3)2-2	8.918e-11	2.668e-11	-10.050	-10.574	-0.524	(0)
CdOH+	4.391e-11	3.248e-11	-10.357	-10.488	-0.131	(0)
Cd(SO4)2-2	3.393e-11	1.015e-11	-10.469	-10.993	-0.524	-104.89
CdH2PO4+	2.466e-11	1.823e-11	-10.608	-10.739	-0.131	(0)
Cd(HPO4)2-2	1.725e-11	5.161e-12	-10.763	-11.287	-0.524	(0)
Cd(Nta)2-4	1.497e-12	1.200e-14	-11.825	-13.921	-2.096	(0)
Cd(OH)2	3.266e-14	3.452e-14	-13.486	-13.462	0.024	(0)
Cd2OH+3	4.739e-17	3.138e-18	-16.324	-17.503	-1.179	(0)
Cd(OH)3-	1.037e-19	7.666e-20	-18.984	-19.115	-0.131	(0)
Cd(OH)4-2	4.520e-26	1.352e-26	-25.345	-25.869	-0.524	(0)
Trizma2.2Cd+2	1.767e-39	5.287e-40	-38.753	-39.277	-0.524	(0)
Cl	1.656e-01					
Cl-	1.656e-01	1.163e-01	-0.781	-0.935	-0.154	18.50
CdCl+	2.962e-07	2.191e-07	-6.528	-6.659	-0.131	7.92
CdCl2	1.005e-07	1.062e-07	-6.998	-6.974	0.024	23.14
CdCl3-	1.053e-08	7.790e-09	-7.977	-8.108	-0.131	69.75
CdOHCl	1.694e-09	1.791e-09	-8.771	-8.747	0.024	(0)
H(0)	0.000e+00					
H2	0.000e+00	0.000e+00	-44.309	-44.285	0.024	28.61
K	3.313e-03					
K+	3.303e-03	2.304e-03	-2.481	-2.637	-0.156	9.38
KSO4-	8.887e-06	6.532e-06	-5.051	-5.185	-0.134	34.54
KHPO4-	1.397e-06	1.027e-06	-5.855	-5.989	-0.134	38.82
KNta-2	1.299e-12	3.887e-13	-11.886	-12.410	-0.524	(0)
Mg	1.240e-03					
Mg+2	1.007e-03	3.317e-04	-2.997	-3.479	-0.482	-20.86
MgHCO3+	1.366e-04	9.625e-05	-3.865	-4.017	-0.152	5.73
MgHPO4	5.315e-05	5.618e-05	-4.274	-4.250	0.024	(0)
MgSO4	2.968e-05	3.137e-05	-4.528	-4.504	0.024	5.84
MgCO3	6.950e-06	7.345e-06	-5.158	-5.134	0.024	-17.09
MgPO4-	3.600e-06	2.646e-06	-5.444	-5.577	-0.134	(0)
MgH2PO4+	2.712e-06	1.993e-06	-5.567	-5.700	-0.134	(0)
MgOH+	3.158e-08	2.384e-08	-7.501	-7.623	-0.122	(0)
MgNta-	1.545e-08	1.142e-08	-7.811	-7.942	-0.131	(0)
Na(1)	2.469e-01					
Na+	2.439e-01	1.803e-01	-0.613	-0.744	-0.131	-0.88
NaHCO3	2.379e-03	2.514e-03	-2.624	-2.600	0.024	1.80
NaSO4-	4.959e-04	3.645e-04	-3.305	-3.438	-0.134	17.97
NaHPO4-	1.093e-04	8.031e-05	-3.961	-4.095	-0.134	50.71
NaCO3-	1.053e-04	7.790e-05	-3.977	-4.108	-0.131	0.53
NaNta-2	4.046e-10	1.211e-10	-9.393	-9.917	-0.524	(0)
NaOH	3.417e-18	3.612e-18	-17.466	-17.442	0.024	(0)
Nta	5.081e-07					
H(Nta)-2	4.316e-07	4.316e-08	-6.365	-7.365	-1.000	(0)
CaNta-	5.297e-08	3.918e-08	-7.276	-7.407	-0.131	(0)
MgNta-	1.545e-08	1.142e-08	-7.811	-7.942	-0.131	(0)
CdNta-	6.968e-09	5.154e-09	-8.157	-8.288	-0.131	(0)
Nta-3	6.856e-10	4.540e-11	-9.164	-10.343	-1.179	(0)
NaNta-2	4.046e-10	1.211e-10	-9.393	-9.917	-0.524	(0)
H2(Nta)-	3.366e-12	1.892e-12	-11.473	-11.723	-0.250	(0)

Cd(Nta)2-4		1.497e-12	1.200e-14	-11.825	-13.921	-2.096	(0)
KNta-2		1.299e-12	3.887e-13	-11.886	-12.410	-0.524	(0)
Ca(Nta)2-4		5.479e-14	4.391e-16	-13.261	-15.357	-2.096	(0)
H3(Nta)		9.485e-18	9.485e-18	-17.023	-17.023	0.000	(0)
H4(Nta)+		8.454e-24	4.754e-24	-23.073	-23.323	-0.250	(0)
O(0)	2.886e-04						
O2		1.443e-04	1.525e-04	-3.841	-3.817	0.024	30.40
P	1.280e-03						
HPO4-2		8.182e-04	2.285e-04	-3.087	-3.641	-0.554	8.44
H2PO4-		2.509e-04	1.844e-04	-3.600	-3.734	-0.134	33.91
NaHPO4-		1.093e-04	8.031e-05	-3.961	-4.095	-0.134	50.71
MgHPO4		5.315e-05	5.618e-05	-4.274	-4.250	0.024	(0)
CaHPO4		3.671e-05	3.880e-05	-4.435	-4.411	0.024	(0)
MgPO4-		3.600e-06	2.646e-06	-5.444	-5.577	-0.134	(0)
MgH2PO4+		2.712e-06	1.993e-06	-5.567	-5.700	-0.134	(0)
CaPO4-		2.492e-06	1.832e-06	-5.603	-5.737	-0.134	(0)
CaH2PO4+		1.989e-06	1.462e-06	-5.701	-5.835	-0.134	(0)
KHPO4-		1.397e-06	1.027e-06	-5.855	-5.989	-0.134	38.82
PO4-3		4.802e-08	2.055e-09	-7.319	-8.687	-1.369	-19.58
CdHPO4		2.691e-09	2.844e-09	-8.570	-8.546	0.024	(0)
H3PO4		1.288e-09	1.361e-09	-8.890	-8.866	0.024	47.41
CdH2PO4+		2.466e-11	1.823e-11	-10.608	-10.739	-0.131	(0)
Cd(HPO4)2-2		1.725e-11	5.161e-12	-10.763	-11.287	-0.524	(0)
S(6)	2.032e-03						
SO4-2		1.477e-03	4.034e-04	-2.831	-3.394	-0.564	16.24
NaSO4-		4.959e-04	3.645e-04	-3.305	-3.438	-0.134	17.97
MgSO4		2.968e-05	3.137e-05	-4.528	-4.504	0.024	5.84
CaSO4		2.102e-05	2.222e-05	-4.677	-4.653	0.024	7.50
KSO4-		8.887e-06	6.532e-06	-5.051	-5.185	-0.134	34.54
HSO4-		2.658e-09	1.966e-09	-8.575	-8.706	-0.131	40.69
CdSO4		2.172e-09	2.295e-09	-8.663	-8.639	0.024	78.05
Cd(SO4)2-2		3.393e-11	1.015e-11	-10.469	-10.993	-0.524	-104.89
CaHSO4+		9.897e-12	7.320e-12	-11.004	-11.135	-0.131	(0)
Trizma	1.524e-02						
TrizmaH+		1.524e-02	1.127e-02	-1.817	-1.948	-0.131	(0)
Trizma		1.391e-17	1.470e-17	-16.857	-16.833	0.024	(0)
Trizma2.2Cd+2		1.767e-39	5.287e-40	-38.753	-39.277	-0.524	(0)

------------------------------ Saturation indices------------------------------

Phase	SI**	log IAP	log K(298 K, 1 atm)	
Anhydrite	-2.63	-6.90	-4.28	CaSO4
Aragonite	0.19	-8.14	-8.34	CaCO3
Calcite	0.34	-8.14	-8.48	CaCO3
Cd(OH)2	-6.76	6.89	13.65	Cd(OH)2
Cd3(PO4)2	-7.89	-40.49	-32.60	Cd3(PO4)2
CdSO4	-11.00	-11.10	-0.10	CdSO4
CO2(g)	-1.08	-2.55	-1.47	CO2
Dolomite	0.83	-16.26	-17.08	CaMg(CO3)2
Gypsum	-2.33	-6.91	-4.58	CaSO4:2H2O
H2(g)	-41.18	-44.29	-3.10	H2
H2O(g)	-1.51	-0.00	1.50	H2O
Halite	-3.25	-1.68	1.57	NaCl
Hydroxyapatite	4.15	0.73	-3.42	Ca5(PO4)3OH
O2(g)	-0.92	-3.82	-2.89	O2
Otavite	-0.24	-12.34	-12.10	CdCO3
Sylvite	-4.47	-3.57	0.90	KCl

**For a gas, SI = log10(fugacity). Fugacity = pressure * phi / 1 atm.
For ideal gases, phi = 1.

End of simulation.

Reading input data for simulation 2.

End of Run after 82.744 Seconds.

PHREEQC output file for Cd^{2+}+EDTA in tris

Input file: cd-tris-edta.txt
Output file: cd-tris-edta.txt.
Database file: C:\Users\genek\Documents\Research\phreeqc-3.7.3-15968-x64\database\phreeqc.dat

```
------------------
Reading data base.
------------------

        SOLUTION_MASTER_SPECIES
        SOLUTION_SPECIES
        PHASES
        EXCHANGE_MASTER_SPECIES
        EXCHANGE_SPECIES
        SURFACE_MASTER_SPECIES
        SURFACE_SPECIES
        RATES
        END
------------------------------------
Reading input data for simulation 1.
------------------------------------

        SOLUTION 1 Test2
                temp 25.0
                pH 7.3
                pe 13.7848
                redox O(-2)/O(0)
                units mmol/L
                Ca 1.198
                K  3.26
                Na 243
                Mg 1.22
                Cl 163
                S(6) 2 P
                1.26
                O(0) 0.284
                C(4) 38.63
                Cd 0.0005
                Edta 0.0005
                Trizma 15
        END
-------------------------------------------
Beginning of initial solution calculations.
-------------------------------------------

Initial solution 1.             Test2

-----------------------------                Solution composition------------------------------

        Elements              Molality       Moles

        C(4)                 3.926e-02   3.926e-02
        Ca                   1.217e-03   1.217e-03
        Cd                   5.081e-07   5.081e-07
        Cl                   1.656e-01   1.656e-01
        Edta                 5.081e-07   5.081e-07
        K                    3.313e-03   3.313e-03
        Mg                   1.240e-03   1.240e-03
        Na                   2.469e-01   2.469e-01
        O(0)                 2.886e-04   2.886e-04
        P                    1.280e-03   1.280e-03
```

S(6)	2.032e-03	2.032e-03

Trizma	1.524e-02	1.524e-02

----------------------------Description of solution----------------------------

pH	=	7.300
pe	=	13.785
Specific Conductance (µS/cm, 25°C)	=	21158
Density (g/cm³)	=	1.00940
Volume (L)	=	1.00683
Activity of water	=	0.992
Ionic strength (mol/kgw)	=	2.403e-01
Mass of water (kg)	=	1.000e+00
Total alkalinity (eq/kg)	=	2.258e-02
Total CO2 (mol/kg)	=	3.926e-02
Temperature (°C)	=	25.00
Electrical balance (eq)	=	6.160e-02
Percent error, 100*(Cat-|An|)/(Cat+|An|)	=	13.06
Iterations	=	10
Total H	=	1.110656e+02
Total O	=	5.563486e+01

---------------------------------Redox couples---------------------------------

Redox couple	pe	Eh (volts)
O(-2)/O(0)	13.2676	0.7849

----------------------------Distribution of species----------------------------

Species	Molality	Activity	Log Molality	Log Activity	Log Gamma	mole V cm³/mol
OH-	2.895e-07	2.003e-07	-6.538	-6.698	-0.160	-3.41
H+	6.340e-08	5.012e-08	-7.198	-7.300	-0.102	0.00
H2O	5.551e+01	9.920e-01	1.744	-0.004	0.000	18.07
C(4)	3.926e-02					
HCO3-	3.374e-02	2.480e-02	-1.472	-1.606	-0.134	25.67
CO2	2.665e-03	2.817e-03	-2.574	-2.550	0.024	34.43
NaHCO3	2.379e-03	2.514e-03	-2.624	-2.600	0.024	1.80
MgHCO3+	1.366e-04	9.625e-05	-3.865	-4.017	-0.152	5.73
CaHCO3+	1.313e-04	9.797e-05	-3.882	-4.009	-0.127	9.89
NaCO3-	1.053e-04	7.790e-05	-3.977	-4.108	-0.131	0.53
CO3-2	7.953e-05	2.320e-05	-4.099	-4.634	-0.535	-3.43
CaCO3	1.142e-05	1.207e-05	-4.942	-4.918	0.024	-14.60
MgCO3	6.950e-06	7.345e-06	-5.158	-5.134	0.024	-17.09
(CO2)2	1.378e-07	1.457e-07	-6.861	-6.837	0.024	68.87
CdHCO3+	1.841e-08	1.362e-08	-7.735	-7.866	-0.131	(0)
CdCO3	3.028e-10	3.200e-10	-9.519	-9.495	0.024	(0)
Cd(CO3)2-2	7.848e-11	2.348e-11	-10.105	-10.629	-0.524	(0)
Ca	1.217e-03					
Ca+2	1.012e-03	3.096e-04	-2.995	-3.509	-0.514	-17.14
CaHCO3+	1.313e-04	9.797e-05	-3.882	-4.009	-0.127	9.89
CaHPO4	3.670e-05	3.879e-05	-4.435	-4.411	0.024	(0)
CaSO4	2.102e-05	2.221e-05	-4.677	-4.653	0.024	7.50
CaCO3	1.142e-05	1.207e-05	-4.942	-4.918	0.024	-14.60
CaPO4-	2.491e-06	1.831e-06	-5.604	-5.737	-0.134	(0)
CaH2PO4+	1.988e-06	1.461e-06	-5.702	-5.835	-0.134	(0)
Ca(Edta)-2	4.344e-07	4.343e-08	-6.362	-7.362	-1.000	(0)
CaOH+	1.375e-09	1.017e-09	-8.862	-8.993	-0.131	(0)
CaH(Edta)-	1.169e-11	6.574e-12	-10.932	-11.182	-0.250	(0)
CaHSO4+	9.894e-12	7.318e-12	-11.005	-11.136	-0.131	(0)

Cd	5.081e-07					
CdCl+	2.607e-07	1.928e-07	-6.584	-6.715	-0.131	7.92
CdCl2	8.841e-08	9.344e-08	-7.053	-7.029	0.024	23.14
Cd(Edta)-2	6.708e-08	6.707e-09	-7.173	-8.173	-1.000	(0)
Cd+2	5.803e-08	1.736e-08	-7.236	-7.760	-0.524	-17.84
CdHCO3+	1.841e-08	1.362e-08	-7.735	-7.866	-0.131	(0)
CdCl3-	9.269e-09	6.855e-09	-8.033	-8.164	-0.131	69.75
CdHPO4	2.368e-09	2.503e-09	-8.626	-8.602	0.024	(0)
CdSO4	1.911e-09	2.020e-09	-8.719	-8.695	0.024	78.05
CdOHCl	1.491e-09	1.576e-09	-8.826	-8.802	0.024	(0)
CdCO3	3.028e-10	3.200e-10	-9.519	-9.495	0.024	(0)
Cd(CO3)2-2	7.848e-11	2.348e-11	-10.105	-10.629	-0.524	(0)
CdOH+	3.864e-11	2.858e-11	-10.413	-10.544	-0.131	(0)
Cd(SO4)2-2	2.986e-11	8.934e-12	-10.525	-11.049	-0.524	-104.89
CdH2PO4+	2.170e-11	1.605e-11	-10.664	-10.795	-0.131	(0)
Cd(HPO4)2-2	1.518e-11	4.542e-12	-10.819	-11.343	-0.524	(0)
CdH(Edta)-	3.772e-13	2.121e-13	-12.423	-12.673	-0.250	(0)
Cd(OH)2	2.874e-14	3.038e-14	-13.541	-13.517	0.024	(0)
Cd2OH+3	3.671e-17	2.430e-18	-16.435	-17.614	-1.179	(0)
Cd(OH)3-	9.122e-20	6.746e-20	-19.040	-19.171	-0.131	(0)
Cd(OH)4-2	3.977e-26	1.190e-26	-25.400	-25.924	-0.524	(0)
Trizma2.2Cd+2	1.555e-39	4.653e-40	-38.808	-39.332	-0.524	(0)
Cl	1.656e-01					
Cl-	1.656e-01	1.163e-01	-0.781	-0.935	-0.154	18.50
CdCl+	2.607e-07	1.928e-07	-6.584	-6.715	-0.131	7.92
CdCl2	8.841e-08	9.344e-08	-7.053	-7.029	0.024	23.14
CdCl3-	9.269e-09	6.855e-09	-8.033	-8.164	-0.131	69.75
CdOHCl	1.491e-09	1.576e-09	-8.826	-8.802	0.024	(0)
Edta	5.081e-07					
Ca(Edta)-2	4.344e-07	4.343e-08	-6.362	-7.362	-1.000	(0)
Cd(Edta)-2	6.708e-08	6.707e-09	-7.173	-8.173	-1.000	(0)
Mg(Edta)-2	6.573e-09	6.572e-10	-8.182	-9.182	-1.000	(0)
H(Edta)-3	4.218e-11	2.371e-13	-10.375	-12.625	-2.250	(0)
CaH(Edta)-	1.169e-11	6.574e-12	-10.932	-11.182	-0.250	(0)
MgH(Edta)-	1.471e-12	8.274e-13	-11.832	-12.082	-0.250	(0)
Na(Edta)-3	8.572e-13	4.818e-15	-12.067	-14.317	-2.250	(0)
CdH(Edta)-	3.772e-13	2.121e-13	-12.423	-12.673	-0.250	(0)
H2(Edta)-2	2.229e-13	2.228e-14	-12.652	-13.652	-1.000	(0)
Edta-4	6.653e-15	5.333e-17	-14.177	-16.273	-2.096	(0)
K(Edta)-3	1.096e-15	6.159e-18	-14.960	-17.210	-2.250	(0)
H3(Edta)-	2.612e-18	1.469e-18	-17.583	-17.833	-0.250	(0)
H4(Edta)	1.064e-23	1.064e-23	-22.973	-22.973	0.000	(0)
H5(Edta)+	2.999e-29	1.686e-29	-28.523	-28.773	-0.250	(0)
H(0)	0.000e+00					
H2	0.000e+00	0.000e+00	-44.309	-44.285	0.024	28.61
K	3.313e-03					
K+	3.303e-03	2.304e-03	-2.481	-2.637	-0.156	9.38
KSO4-	8.887e-06	6.532e-06	-5.051	-5.185	-0.134	34.54
KHPO4-	1.397e-06	1.027e-06	-5.855	-5.989	-0.134	38.82
K(Edta)-3	1.096e-15	6.159e-18	-14.960	-17.210	-2.250	(0)
Mg	1.240e-03					
Mg+2	1.007e-03	3.317e-04	-2.997	-3.479	-0.482	-20.86
MgHCO3+	1.366e-04	9.625e-05	-3.865	-4.017	-0.152	5.73
MgHPO4	5.316e-05	5.618e-05	-4.274	-4.250	0.024	(0)
MgSO4	2.968e-05	3.137e-05	-4.528	-4.504	0.024	5.84
MgCO3	6.950e-06	7.345e-06	-5.158	-5.134	0.024	-17.09
MgPO4-	3.600e-06	2.646e-06	-5.444	-5.577	-0.134	(0)
MgH2PO4+	2.712e-06	1.993e-06	-5.567	-5.700	-0.134	(0)
MgOH+	3.158e-08	2.384e-08	-7.501	-7.623	-0.122	(0)
Mg(Edta)-2	6.573e-09	6.572e-10	-8.182	-9.182	-1.000	(0)
MgH(Edta)-	1.471e-12	8.274e-13	-11.832	-12.082	-0.250	(0)

Na(1)	2.469e-01					
Na+	2.439e-01	1.803e-01	-0.613	-0.744	-0.131	-0.88
NaHCO3	2.379e-03	2.514e-03	-2.624	-2.600	0.024	1.80
NaSO4-	4.959e-04	3.645e-04	-3.305	-3.438	-0.134	17.97
NaHPO4-	1.093e-04	8.031e-05	-3.961	-4.095	-0.134	50.71
NaCO3-	1.053e-04	7.790e-05	-3.977	-4.108	-0.131	0.53
Na(Edta)-3	8.572e-13	4.818e-15	-12.067	-14.317	-2.250	(0)
NaOH	3.417e-18	3.612e-18	-17.466	-17.442	0.024	(0)
O(0)	2.886e-04					
O2	1.443e-04	1.525e-04	-3.841	-3.817	0.024	30.40
P	1.280e-03					
HPO4-2	8.182e-04	2.285e-04	-3.087	-3.641	-0.554	8.44
H2PO4-	2.509e-04	1.844e-04	-3.600	-3.734	-0.134	33.91
NaHPO4-	1.093e-04	8.031e-05	-3.961	-4.095	-0.134	50.71
MgHPO4	5.316e-05	5.618e-05	-4.274	-4.250	0.024	(0)
CaHPO4	3.670e-05	3.879e-05	-4.435	-4.411	0.024	(0)
MgPO4-	3.600e-06	2.646e-06	-5.444	-5.577	-0.134	(0)
MgH2PO4+	2.712e-06	1.993e-06	-5.567	-5.700	-0.134	(0)
CaPO4-	2.491e-06	1.831e-06	-5.604	-5.737	-0.134	(0)
CaH2PO4+	1.988e-06	1.461e-06	-5.702	-5.835	-0.134	(0)
KHPO4-	1.397e-06	1.027e-06	-5.855	-5.989	-0.134	38.82
PO4-3	4.802e-08	2.055e-09	-7.319	-8.687	-1.369	-19.58
CdHPO4	2.368e-09	2.503e-09	-8.626	-8.602	0.024	(0)
H3PO4	1.288e-09	1.361e-09	-8.890	-8.866	0.024	47.41
CdH2PO4+	2.170e-11	1.605e-11	-10.664	-10.795	-0.131	(0)
Cd(HPO4)2-2	1.518e-11	4.542e-12	-10.819	-11.343	-0.524	(0)
S(6)	2.032e-03					
SO4-2	1.477e-03	4.034e-04	-2.831	-3.394	-0.564	16.24
NaSO4-	4.959e-04	3.645e-04	-3.305	-3.438	-0.134	17.97
MgSO4	2.968e-05	3.137e-05	-4.528	-4.504	0.024	5.84
CaSO4	2.102e-05	2.221e-05	-4.677	-4.653	0.024	7.50
KSO4-	8.887e-06	6.532e-06	-5.051	-5.185	-0.134	34.54
HSO4-	2.658e-09	1.966e-09	-8.575	-8.706	-0.131	40.69
CdSO4	1.911e-09	2.020e-09	-8.719	-8.695	0.024	78.05
Cd(SO4)2-2	2.986e-11	8.934e-12	-10.525	-11.049	-0.524	-104.89
CaHSO4+	9.894e-12	7.318e-12	-11.005	-11.136	-0.131	(0)
Trizma	1.524e-02					
TrizmaH+	1.524e-02	1.127e-02	-1.817	-1.948	-0.131	(0)
Trizma	1.391e-17	1.470e-17	-16.857	-16.833	0.024	(0)
Trizma2.2Cd+2	1.555e-39	4.653e-40	-38.808	-39.332	-0.524	(0)

------------------------------ Saturation indices-------------------------------

Phase	SI**	log IAP	log K(298 K, 1 atm)	
Anhydrite	-2.63	-6.90	-4.28	CaSO4
Aragonite	0.19	-8.14	-8.34	CaCO3
Calcite	0.34	-8.14	-8.48	CaCO3
Cd(OH)2	-6.82	6.83	13.65	Cd(OH)2
Cd3(PO4)2	-8.06	-40.66	-32.60	Cd3(PO4)2
CdSO4	-11.05	-11.15	-0.10	CdSO4
CO2(g)	-1.08	-2.55	-1.47	CO2
Dolomite	0.83	-16.26	-17.08	CaMg(CO3)2
Gypsum	-2.33	-6.91	-4.58	CaSO4:2H2O
H2(g)	-41.18	-44.29	-3.10	H2
H2O(g)	-1.51	-0.00	1.50	H2O
Halite	-3.25	-1.68	1.57	NaCl
Hydroxyapatite	4.15	0.73	-3.42	Ca5(PO4)3OH
O2(g)	-0.92	-3.82	-2.89	O2
Otavite	-0.29	-12.39	-12.10	CdCO3
Sylvite	-4.47	-3.57	0.90	KCl

**For a gas, SI = log10(fugacity). Fugacity = pressure * phi / 1 atm.
For ideal gases, phi = 1.

End of simulation.

Reading input data for simulation 2.

End of Run after 12.222 Seconds.

PHREEQC output file for Cd^{2+}+DMSA in tris

Input file: cd-tris-dmsa
Output file: cd-tris-dmsa.out
Database file: C:\Users\genek\Documents\Research\phreeqc-3.7.3-15968-x64\database\phreeqc.dat

----
Reading data base.

SOLUTION_MASTER_SPECIES
SOLUTION_SPECIES
PHASES
EXCHANGE_MASTER_SPECIES
EXCHANGE_SPECIES
SURFACE_MASTER_SPECIES
SURFACE_SPECIES
RATES
END

Reading input data for simulation 1.

SOLUTION 1 Test2
temp 25.0
pH 7.3
pe 13.7848
redox O(-2)/O(0)
units mmol/L
Ca 1.198
K 3.26
Na 243
Mg 1.22
Cl 163
S(6) 2 P
1.26
O(0) 0.284
C(4) 38.63 Cd
0.0005 Dmsa
0.0005 Trizma
15
END

Beginning of initial solution calculations.

Initial solution 1. Test2

-----------------------------Solution composition------------------------------

Elements	Molality	Moles
C(4)	3.926e-02	3.926e-02
Ca	1.217e-03	1.217e-03
Cd	5.081e-07	5.081e-07
Cl	1.656e-01	1.656e-01
Dmsa	5.081e-07	5.081e-07
K	3.313e-03	3.313e-03
Mg	1.240e-03	1.240e-03
Na	2.469e-01	2.469e-01
O(0)	2.886e-04	2.886e-04
P	1.280e-03	1.280e-03
S(6)	2.032e-03	2.032e-03

---------------------------- Description of solution----------------------------

pH	=	7.300
pe	=	13.785
Specific Conductance (µS/cm, 25°C)	=	21158
Density (g/cm³)	=	1.00940
Volume (L)	=	1.00683
Activity of water	=	0.992
Ionic strength (mol/kgw)	=	2.403e-01
Mass of water (kg)	=	1.000e+00
Total alkalinity (eq/kg)	=	2.258e-02
Total CO2 (mol/kg)	=	3.926e-02
Temperature (°C)	=	25.00
Electrical balance (eq)	=	6.160e-02
Percent error, 100*(Cat-|An|)/(Cat+|An|)	=	13.06
Iterations	=	14
Total H	=	1.110656e+02
Total O	=	5.563486e+01

--------------------------------- Redox couples---------------------------------

Redox couple	pe	Eh (volts)
O(-2)/O(0)	13.2676	0.7849

---------------------------- Distribution of species----------------------------

Species	Molality	Activity	Log Molality	Log Activity	Log Gamma	mole V cm³/mol
OH-	2.895e-07	2.003e-07	-6.538	-6.698	-0.160	-3.41
H+	6.340e-08	5.012e-08	-7.198	-7.300	-0.102	0.00
H2O	5.551e+01	9.920e-01	1.744	-0.004	0.000	18.07
C(4)	3.926e-02					
HCO3-	3.374e-02	2.480e-02	-1.472	-1.606	-0.134	25.67
CO2	2.665e-03	2.817e-03	-2.574	-2.550	0.024	34.43
NaHCO3	2.379e-03	2.514e-03	-2.624	-2.600	0.024	1.80
MgHCO3+	1.366e-04	9.625e-05	-3.865	-4.017	-0.152	5.73
CaHCO3+	1.313e-04	9.800e-05	-3.882	-4.009	-0.127	9.89
NaCO3-	1.053e-04	7.790e-05	-3.977	-4.108	-0.131	0.53
CO3-2	7.953e-05	2.320e-05	-4.099	-4.634	-0.535	-3.43
CaCO3	1.142e-05	1.207e-05	-4.942	-4.918	0.024	-14.60
MgCO3	6.950e-06	7.345e-06	-5.158	-5.134	0.024	-17.09
(CO2)2	1.378e-07	1.457e-07	-6.861	-6.837	0.024	68.87
CdHCO3+	1.412e-09	1.044e-09	-8.850	-8.981	-0.131	(0)
CdCO3	2.322e-11	2.455e-11	-10.634	-10.610	0.024	(0)
Cd(CO3)2-2	6.020e-12	1.801e-12	-11.220	-11.744	-0.524	(0)
Ca	1.217e-03					
Ca+2	1.012e-03	3.098e-04	-2.995	-3.509	-0.514	-17.14
CaHCO3+	1.313e-04	9.800e-05	-3.882	-4.009	-0.127	9.89
CaHPO4	3.671e-05	3.880e-05	-4.435	-4.411	0.024	(0)
CaSO4	2.102e-05	2.222e-05	-4.677	-4.653	0.024	7.50
CaCO3	1.142e-05	1.207e-05	-4.942	-4.918	0.024	-14.60
CaPO4-	2.492e-06	1.832e-06	-5.603	-5.737	-0.134	(0)
CaH2PO4+	1.989e-06	1.462e-06	-5.701	-5.835	-0.134	(0)
CaOH+	1.376e-09	1.017e-09	-8.861	-8.992	-0.131	(0)
CaHSO4+	9.898e-12	7.320e-12	-11.004	-11.135	-0.131	(0)
Cd	5.081e-07					
CdDmsa-2	4.518e-07	1.352e-07	-6.345	-6.869	-0.524	(0)

		HCdDmsa-	2.249e-08	1.663e-08	-7.648	-7.779	-0.131	(0)
		CdCl+	1.999e-08	1.479e-08	-7.699	-7.830	-0.131	7.92
		CdCl2	6.781e-09	7.167e-09	-8.169	-8.145	0.024	23.14
		Cd+2	4.451e-09	1.332e-09	-8.352	-8.876	-0.524	-17.84
		CdHCO3+	1.412e-09	1.044e-09	-8.850	-8.981	-0.131	(0)
		CdCl3-	7.109e-10	5.258e-10	-9.148	-9.279	-0.131	69.75
		CdHPO4	1.816e-10	1.920e-10	-9.741	-9.717	0.024	(0)
		CdSO4	1.466e-10	1.549e-10	-9.834	-9.810	0.024	78.05
		CdOHCl	1.144e-10	1.209e-10	-9.942	-9.918	0.024	(0)
		CdCO3	2.322e-11	2.455e-11	-10.634	-10.610	0.024	(0)
		Cd(CO3)2-2	6.020e-12	1.801e-12	-11.220	-11.744	-0.524	(0)
		CdOH+	2.964e-12	2.192e-12	-11.528	-11.659	-0.131	(0)
		Cd(SO4)2-2	2.290e-12	6.853e-13	-11.640	-12.164	-0.524	-104.89
		CdH2PO4+	1.664e-12	1.231e-12	-11.779	-11.910	-0.131	(0)
		Cd(HPO4)2-2	1.164e-12	3.484e-13	-11.934	-12.458	-0.524	(0)
		Cd(OH)2	2.205e-15	2.330e-15	-14.657	-14.633	0.024	(0)
		H3CdDmsa+	9.593e-18	7.095e-18	-17.018	-17.149	-0.131	(0)
		Cd2OH+3	2.159e-19	1.430e-20	-18.666	-19.845	-1.179	(0)
		Cd(OH)3-	6.996e-21	5.175e-21	-20.155	-20.286	-0.131	(0)
		Cd(OH)4-2	3.051e-27	9.128e-28	-26.516	-27.040	-0.524	(0)
		Trizma2.2Cd+2	1.193e-40	0.000e+00	-39.923	-40.447	-0.524	(0)
Cl	1.656e-01							
		Cl-	1.656e-01	1.163e-01	-0.781	-0.935	-0.154	18.50
		CdCl+	1.999e-08	1.479e-08	-7.699	-7.830	-0.131	7.92
		CdCl2	6.781e-09	7.167e-09	-8.169	-8.145	0.024	23.14
		CdCl3-	7.109e-10	5.258e-10	-9.148	-9.279	-0.131	69.75
		CdOHCl	1.144e-10	1.209e-10	-9.942	-9.918	0.024	(0)
Dmsa	5.081e-07							
		CdDmsa-2	4.518e-07	1.352e-07	-6.345	-6.869	-0.524	(0)
		H2Dmsa-2	3.316e-08	9.921e-09	-7.479	-8.003	-0.524	(0)
		HCdDmsa-	2.249e-08	1.663e-08	-7.648	-7.779	-0.131	(0)
		HDmsa-3	6.693e-10	4.431e-11	-9.174	-10.353	-1.179	(0)
		H3Dmsa-	1.809e-12	1.338e-12	-11.742	-11.873	-0.131	(0)
		Dmsa-4	9.832e-14	7.880e-16	-13.007	-15.103	-2.096	(0)
		H4Dmsa	3.255e-17	3.440e-17	-16.487	-16.463	0.024	(0)
		H3CdDmsa+	9.593e-18	7.095e-18	-17.018	-17.149	-0.131	(0)
H(0)	0.000e+00							
		H2	0.000e+00	0.000e+00	-44.309	-44.285	0.024	28.61
K	3.313e-03							
		K+	3.303e-03	2.304e-03	-2.481	-2.637	-0.156	9.38
		KSO4-	8.887e-06	6.532e-06	-5.051	-5.185	-0.134	34.54
		KHPO4-	1.397e-06	1.027e-06	-5.855	-5.989	-0.134	38.82
Mg	1.240e-03							
		Mg+2	1.007e-03	3.317e-04	-2.997	-3.479	-0.482	-20.86
		MgHCO3+	1.366e-04	9.625e-05	-3.865	-4.017	-0.152	5.73
		MgHPO4	5.316e-05	5.618e-05	-4.274	-4.250	0.024	(0)
		MgSO4	2.968e-05	3.137e-05	-4.528	-4.504	0.024	5.84
		MgCO3	6.950e-06	7.345e-06	-5.158	-5.134	0.024	-17.09
		MgPO4-	3.600e-06	2.646e-06	-5.444	-5.577	-0.134	(0)
		MgH2PO4+	2.712e-06	1.993e-06	-5.567	-5.700	-0.134	(0)
		MgOH+	3.158e-08	2.384e-08	-7.501	-7.623	-0.122	(0)
Na(1)	2.469e-01							
		Na+	2.439e-01	1.803e-01	-0.613	-0.744	-0.131	-0.88
		NaHCO3	2.379e-03	2.514e-03	-2.624	-2.600	0.024	1.80
		NaSO4-	4.959e-04	3.645e-04	-3.305	-3.438	-0.134	17.97
		NaHPO4-	1.093e-04	8.031e-05	-3.961	-4.095	-0.134	50.71
		NaCO3-	1.053e-04	7.790e-05	-3.977	-4.108	-0.131	0.53
		NaOH	3.417e-18	3.612e-18	-17.466	-17.442	0.024	(0)
O(0)	2.886e-04							
		O2	1.443e-04	1.525e-04	-3.841	-3.817	0.024	30.40
P	1.280e-03							

HPO4-2	8.182e-04	2.285e-04	-3.087	-3.641	-0.554	8.44
H2PO4-	2.509e-04	1.844e-04	-3.600	-3.734	-0.134	33.91
NaHPO4-	1.093e-04	8.031e-05	-3.961	-4.095	-0.134	50.71
MgHPO4	5.316e-05	5.618e-05	-4.274	-4.250	0.024	(0)
CaHPO4	3.671e-05	3.880e-05	-4.435	-4.411	0.024	(0)
MgPO4-	3.600e-06	2.646e-06	-5.444	-5.577	-0.134	(0)
MgH2PO4+	2.712e-06	1.993e-06	-5.567	-5.700	-0.134	(0)
CaPO4-	2.492e-06	1.832e-06	-5.603	-5.737	-0.134	(0)
CaH2PO4+	1.989e-06	1.462e-06	-5.701	-5.835	-0.134	(0)
KHPO4-	1.397e-06	1.027e-06	-5.855	-5.989	-0.134	38.82
PO4-3	4.802e-08	2.055e-09	-7.319	-8.687	-1.369	-19.58
H3PO4	1.288e-09	1.361e-09	-8.890	-8.866	0.024	47.41
CdHPO4	1.816e-10	1.920e-10	-9.741	-9.717	0.024	(0)
CdH2PO4+	1.664e-12	1.231e-12	-11.779	-11.910	-0.131	(0)
Cd(HPO4)2-2	1.164e-12	3.484e-13	-11.934	-12.458	-0.524	(0)
S(6)	2.032e-03					
SO4-2	1.477e-03	4.034e-04	-2.831	-3.394	-0.564	16.24
NaSO4-	4.959e-04	3.645e-04	-3.305	-3.438	-0.134	17.97
MgSO4	2.968e-05	3.137e-05	-4.528	-4.504	0.024	5.84
CaSO4	2.102e-05	2.222e-05	-4.677	-4.653	0.024	7.50
KSO4-	8.887e-06	6.532e-06	-5.051	-5.185	-0.134	34.54
HSO4-	2.658e-09	1.966e-09	-8.575	-8.706	-0.131	40.69
CdSO4	1.466e-10	1.549e-10	-9.834	-9.810	0.024	78.05
CaHSO4+	9.898e-12	7.320e-12	-11.004	-11.135	-0.131	(0)
Cd(SO4)2-2	2.290e-12	6.853e-13	-11.640	-12.164	-0.524	-104.89
Trizma	1.524e-02					
TrizmaH+	1.524e-02	1.127e-02	-1.817	-1.948	-0.131	(0)
Trizma	1.391e-17	1.470e-17	-16.857	-16.833	0.024	(0)
Trizma2.2Cd+2	1.193e-40	0.000e+00	-39.923	-40.447	-0.524	(0)

-----------------------------Saturation indices-----------------------------

Phase	SI**	log IAP	log K(298 K, 1 atm)	
Anhydrite	-2.63	-6.90	-4.28	CaSO4
Aragonite	0.19	-8.14	-8.34	CaCO3
Calcite	0.34	-8.14	-8.48	CaCO3
Cd(OH)2	-7.93	5.72	13.65	Cd(OH)2
Cd3(PO4)2	-11.40	-44.00	-32.60	Cd3(PO4)2
CdSO4	-12.17	-12.27	-0.10	CdSO4
CO2(g)	-1.08	-2.55	-1.47	CO2
Dolomite	0.83	-16.26	-17.08	CaMg(CO3)2
Gypsum	-2.33	-6.91	-4.58	CaSO4:2H2O
H2(g)	-41.18	-44.29	-3.10	H2
H2O(g)	-1.51	-0.00	1.50	H2O
Halite	-3.25	-1.68	1.57	NaCl
Hydroxyapatite	4.15	0.73	-3.42	Ca5(PO4)3OH
O2(g)	-0.92	-3.82	-2.89	O2
Otavite	-1.41	-13.51	-12.10	CdCO3
Sylvite	-4.47	-3.57	0.90	KCl

**For a gas, SI = log10(fugacity). Fugacity = pressure * phi / 1 atm.
For ideal gases, phi = 1.

End of simulation.

Reading input data for simulation 2.

End of Run after 14.418 Seconds.

PHREEQC output file for Cd2++DPTA in tris

Input file: cd-tris-dtpa
Output file: cd-tris-dtpa.out
Database file: C:\Users\genek\Documents\Research\phreeqc-3.7.3-15968-x64\database\phreeqc.dat

----
Reading data base.

SOLUTION_MASTER_SPECIES
SOLUTION_SPECIES
PHASES
EXCHANGE_MASTER_SPECIES
EXCHANGE_SPECIES
SURFACE_MASTER_SPECIES
SURFACE_SPECIES
RATES
END

Reading input data for simulation 1.

SOLUTION 1 Test2
temp 25.0
pH 7.3
pe 13.7848
redox O(-2)/O(0)
units mmol/L
Ca 1.198
K 3.26
Na 243
Mg 1.22
Cl 163
S(6) 2 P
1.26
O(0) 0.284
C(4) 38.63 Cd
0.0005 Dtpa
0.0005 Trizma
15
END

Beginning of initial solution calculations.

Initial solution 1. Test2

-----------------------------Solution composition------------------------------

Elements	Molality	Moles
C(4)	3.926e-02	3.926e-02
Ca	1.217e-03	1.217e-03
Cd	5.081e-07	5.081e-07
Cl	1.656e-01	1.656e-01
Dtpa	5.081e-07	5.081e-07
K	3.313e-03	3.313e-03
Mg	1.240e-03	1.240e-03
Na	2.469e-01	2.469e-01
O(0)	2.886e-04	2.886e-04
P	1.280e-03	1.280e-03
S(6)	2.032e-03	2.032e-03

Trizma	1.524e-02	1.524e-02

----------------------------Description of solution----------------------------

pH	=	7.300
pe	=	13.785
Specific Conductance (µS/cm, 25°C)	=	21158
Density (g/cm³)	=	1.00940
Volume (L)	=	1.00683
Activity of water	=	0.992
Ionic strength (mol/kgw)	=	2.403e-01
Mass of water (kg)	=	1.000e+00
Total alkalinity (eq/kg)	=	2.258e-02
Total CO2 (mol/kg)	=	3.926e-02
Temperature (°C)	=	25.00
Electrical balance (eq)	=	6.160e-02
Percent error, 100*(Cat-|An|)/(Cat+|An|)	=	13.05
Iterations	=	10
Total H	=	1.110656e+02
Total O	=	5.563486e+01

---------------------------------Redox couples---------------------------------

Redox couple	pe	Eh (volts)
O(-2)/O(0)	13.2676	0.7849

----------------------------Distribution of species----------------------------

Species	Molality	Activity	Log Molality	Log Activity	Log Gamma	mole V cm³/mol
OH-	2.895e-07	2.003e-07	-6.538	-6.698	-0.160	-3.41
H+	6.340e-08	5.012e-08	-7.198	-7.300	-0.102	0.00
H2O	5.551e+01	9.920e-01	1.744	-0.004	0.000	18.07
C(4)	3.926e-02					
HCO3-	3.374e-02	2.480e-02	-1.472	-1.606	-0.134	25.67
CO2	2.665e-03	2.817e-03	-2.574	-2.550	0.024	34.43
NaHCO3	2.379e-03	2.514e-03	-2.624	-2.600	0.024	1.80
MgHCO3+	1.366e-04	9.625e-05	-3.865	-4.017	-0.152	5.73
CaHCO3+	1.313e-04	9.800e-05	-3.882	-4.009	-0.127	9.89
NaCO3-	1.053e-04	7.790e-05	-3.977	-4.108	-0.131	0.53
CO3-2	7.953e-05	2.320e-05	-4.099	-4.634	-0.535	-3.43
CaCO3	1.142e-05	1.207e-05	-4.942	-4.918	0.024	-14.60
MgCO3	6.950e-06	7.345e-06	-5.158	-5.134	0.024	-17.09
(CO2)2	1.378e-07	1.457e-07	-6.861	-6.837	0.024	68.87
CdHCO3+	1.193e-10	8.825e-11	-9.923	-10.054	-0.131	(0)
CdCO3	1.963e-12	2.074e-12	-11.707	-11.683	0.024	(0)
Cd(CO3)2-2	5.087e-13	1.522e-13	-12.294	-12.818	-0.524	(0)
Ca	1.217e-03					
Ca+2	1.012e-03	3.098e-04	-2.995	-3.509	-0.514	-17.14
CaHCO3+	1.313e-04	9.800e-05	-3.882	-4.009	-0.127	9.89
CaHPO4	3.671e-05	3.880e-05	-4.435	-4.411	0.024	(0)
CaSO4	2.102e-05	2.222e-05	-4.677	-4.653	0.024	7.50
CaCO3	1.142e-05	1.207e-05	-4.942	-4.918	0.024	-14.60
CaPO4-	2.492e-06	1.832e-06	-5.603	-5.737	-0.134	(0)
CaH2PO4+	1.989e-06	1.462e-06	-5.701	-5.835	-0.134	(0)
Ca(Dtpa)-3	2.680e-09	1.775e-10	-8.572	-9.751	-1.179	(0)
CaOH+	1.376e-09	1.017e-09	-8.861	-8.992	-0.131	(0)
HCa(Dtpa)-2	3.335e-11	9.979e-12	-10.477	-11.001	-0.524	(0)
CaHSO4+	9.898e-12	7.320e-12	-11.004	-11.135	-0.131	(0)

Ca2(Dtpa)-	5.945e-12	4.397e-12	-11.226	-11.357	-0.131	(0)
**Cd**	5.081e-07					
Cd(Dtpa)-3	5.052e-07	3.345e-08	-6.297	-7.476	-1.179	(0)
CdCl+	1.690e-09	1.250e-09	-8.772	-8.903	-0.131	7.92
CdCl2	5.731e-10	6.057e-10	-9.242	-9.218	0.024	23.14
Cd+2	3.761e-10	1.125e-10	-9.425	-9.949	-0.524	-17.84
CdHCO3+	1.193e-10	8.825e-11	-9.923	-10.054	-0.131	(0)
HCd(Dtpa)-2	7.472e-11	2.236e-11	-10.127	-10.651	-0.524	(0)
CdCl3-	6.008e-11	4.444e-11	-10.221	-10.352	-0.131	69.75
CdHPO4	1.535e-11	1.622e-11	-10.814	-10.790	0.024	(0)
CdSO4	1.239e-11	1.309e-11	-10.907	-10.883	0.024	78.05
CdOHCl	9.666e-12	1.022e-11	-11.015	-10.991	0.024	(0)
CdCO3	1.963e-12	2.074e-12	-11.707	-11.683	0.024	(0)
Cd(CO3)2-2	5.087e-13	1.522e-13	-12.294	-12.818	-0.524	(0)
CdOH+	2.505e-13	1.853e-13	-12.601	-12.732	-0.131	(0)
Cd(SO4)2-2	1.936e-13	5.791e-14	-12.713	-13.237	-0.524	-104.89
CdH2PO4+	1.406e-13	1.040e-13	-12.852	-12.983	-0.131	(0)
Cd(HPO4)2-2	9.839e-14	2.944e-14	-13.007	-13.531	-0.524	(0)
Cd2(Dtpa)-	2.171e-15	1.606e-15	-14.663	-14.794	-0.131	(0)
Cd(OH)2	1.863e-16	1.969e-16	-15.730	-15.706	0.024	(0)
Cd2OH+3	1.542e-21	1.021e-22	-20.812	-21.991	-1.179	(0)
Cd(OH)3-	5.913e-22	4.373e-22	-21.228	-21.359	-0.131	(0)
Cd(OH)4-2	2.578e-28	7.714e-29	-27.589	-28.113	-0.524	(0)
Trizma2.2Cd+2	0.000e+00	0.000e+00	-40.997	-41.521	-0.524	(0)
**Cl**	1.656e-01					
Cl-	1.656e-01	1.163e-01	-0.781	-0.935	-0.154	18.50
CdCl+	1.690e-09	1.250e-09	-8.772	-8.903	-0.131	7.92
CdCl2	5.731e-10	6.057e-10	-9.242	-9.218	0.024	23.14
CdCl3-	6.008e-11	4.444e-11	-10.221	-10.352	-0.131	69.75
CdOHCl	9.666e-12	1.022e-11	-11.015	-10.991	0.024	(0)
**Dtpa**	5.081e-07					
Cd(Dtpa)-3	5.052e-07	3.345e-08	-6.297	-7.476	-1.179	(0)
Ca(Dtpa)-3	2.680e-09	1.775e-10	-8.572	-9.751	-1.179	(0)
Mg(Dtpa)-3	1.224e-10	8.107e-12	-9.912	-11.091	-1.179	(0)
HCd(Dtpa)-2	7.472e-11	2.236e-11	-10.127	-10.651	-0.524	(0)
HCa(Dtpa)-2	3.335e-11	9.979e-12	-10.477	-11.001	-0.524	(0)
HMg(Dtpa)-2	1.267e-11	3.792e-12	-10.897	-11.421	-0.524	(0)
Ca2(Dtpa)-	5.945e-12	4.397e-12	-11.226	-11.357	-0.131	(0)
H2(Dtpa)-3	2.047e-12	1.355e-13	-11.689	-12.868	-1.179	(0)
H(Dtpa)-4	1.691e-12	1.355e-14	-11.772	-13.868	-2.096	(0)
Mg2(Dtpa)-	4.272e-13	3.159e-13	-12.369	-12.500	-0.131	(0)
Dtpa-5	5.601e-14	2.972e-17	-13.252	-16.527	-3.275	(0)
Cd2(Dtpa)-	2.171e-15	1.606e-15	-14.663	-14.794	-0.131	(0)
H3(Dtpa)-2	3.496e-16	1.046e-16	-15.456	-15.980	-0.524	(0)
H4(Dtpa)-	3.259e-21	2.410e-21	-20.487	-20.618	-0.131	(0)
H5(Dtpa)	1.359e-26	1.436e-26	-25.867	-25.843	0.024	(0)
H6(Dtpa)+	3.452e-32	2.553e-32	-31.462	-31.593	-0.131	(0)
H7(Dtpa)+2	2.405e-38	7.196e-39	-37.619	-38.143	-0.524	(0)
H8(Dtpa)+3	0.000e+00	0.000e+00	-44.364	-45.543	-1.179	(0)
**H(0)**	0.000e+00					
H2	0.000e+00	0.000e+00	-44.309	-44.285	0.024	28.61
**K**	3.313e-03					
K+	3.303e-03	2.304e-03	-2.481	-2.637	-0.156	9.38
KSO4-	8.887e-06	6.532e-06	-5.051	-5.185	-0.134	34.54
KHPO4-	1.397e-06	1.027e-06	-5.855	-5.989	-0.134	38.82
**Mg**	1.240e-03					
Mg+2	1.007e-03	3.317e-04	-2.997	-3.479	-0.482	-20.86
MgHCO3+	1.366e-04	9.625e-05	-3.865	-4.017	-0.152	5.73
MgHPO4	5.316e-05	5.618e-05	-4.274	-4.250	0.024	(0)
MgSO4	2.968e-05	3.137e-05	-4.528	-4.504	0.024	5.84
MgCO3	6.950e-06	7.345e-06	-5.158	-5.134	0.024	-17.09

MgPO4-	3.600e-06	2.646e-06	-5.444	-5.577	-0.134	(0)
MgH2PO4+	2.712e-06	1.993e-06	-5.567	-5.700	-0.134	(0)
MgOH+	3.158e-08	2.384e-08	-7.501	-7.623	-0.122	(0)
Mg(Dtpa)-3	1.224e-10	8.107e-12	-9.912	-11.091	-1.179	(0)
HMg(Dtpa)-2	1.267e-11	3.792e-12	-10.897	-11.421	-0.524	(0)
Mg2(Dtpa)-	4.272e-13	3.159e-13	-12.369	-12.500	-0.131	(0)
Na(1)	2.469e-01					
Na+	2.439e-01	1.803e-01	-0.613	-0.744	-0.131	-0.88
NaHCO3	2.379e-03	2.514e-03	-2.624	-2.600	0.024	1.80
NaSO4-	4.959e-04	3.645e-04	-3.305	-3.438	-0.134	17.97
NaHPO4-	1.093e-04	8.031e-05	-3.961	-4.095	-0.134	50.71
NaCO3-	1.053e-04	7.790e-05	-3.977	-4.108	-0.131	0.53
NaOH	3.417e-18	3.612e-18	-17.466	-17.442	0.024	(0)
O(0)	2.886e-04					
O2	1.443e-04	1.525e-04	-3.841	-3.817	0.024	30.40
P	1.280e-03					
HPO4-2	8.182e-04	2.285e-04	-3.087	-3.641	-0.554	8.44
H2PO4-	2.509e-04	1.844e-04	-3.600	-3.734	-0.134	33.91
NaHPO4-	1.093e-04	8.031e-05	-3.961	-4.095	-0.134	50.71
MgHPO4	5.316e-05	5.618e-05	-4.274	-4.250	0.024	(0)
CaHPO4	3.671e-05	3.880e-05	-4.435	-4.411	0.024	(0)
MgPO4-	3.600e-06	2.646e-06	-5.444	-5.577	-0.134	(0)
MgH2PO4+	2.712e-06	1.993e-06	-5.567	-5.700	-0.134	(0)
CaPO4-	2.492e-06	1.832e-06	-5.603	-5.737	-0.134	(0)
CaH2PO4+	1.989e-06	1.462e-06	-5.701	-5.835	-0.134	(0)
KHPO4-	1.397e-06	1.027e-06	-5.855	-5.989	-0.134	38.82
PO4-3	4.802e-08	2.055e-09	-7.319	-8.687	-1.369	-19.58
H3PO4	1.288e-09	1.361e-09	-8.890	-8.866	0.024	47.41
CdHPO4	1.535e-11	1.622e-11	-10.814	-10.790	0.024	(0)
CdH2PO4+	1.406e-13	1.040e-13	-12.852	-12.983	-0.131	(0)
Cd(HPO4)2-2	9.839e-14	2.944e-14	-13.007	-13.531	-0.524	(0)
S(6)	2.032e-03					
SO4-2	1.477e-03	4.034e-04	-2.831	-3.394	-0.564	16.24
NaSO4-	4.959e-04	3.645e-04	-3.305	-3.438	-0.134	17.97
MgSO4	2.968e-05	3.137e-05	-4.528	-4.504	0.024	5.84
CaSO4	2.102e-05	2.222e-05	-4.677	-4.653	0.024	7.50
KSO4-	8.887e-06	6.532e-06	-5.051	-5.185	-0.134	34.54
HSO4-	2.658e-09	1.966e-09	-8.575	-8.706	-0.131	40.69
CdSO4	1.239e-11	1.309e-11	-10.907	-10.883	0.024	78.05
CaHSO4+	9.898e-12	7.320e-12	-11.004	-11.135	-0.131	(0)
Cd(SO4)2-2	1.936e-13	5.791e-14	-12.713	-13.237	-0.524	-104.89
Trizma	1.524e-02					
TrizmaH+	1.524e-02	1.127e-02	-1.817	-1.948	-0.131	(0)
Trizma	1.391e-17	1.470e-17	-16.857	-16.833	0.024	(0)
Trizma2.2Cd+2	0.000e+00	0.000e+00	-40.997	-41.521	-0.524	(0)

------------------------------ Saturation indices------------------------------

Phase	SI**	log IAP	log K(298 K, 1 atm)	
Anhydrite	-2.63	-6.90	-4.28	CaSO4
Aragonite	0.19	-8.14	-8.34	CaCO3
Calcite	0.34	-8.14	-8.48	CaCO3
Cd(OH)2	-9.01	4.64	13.65	Cd(OH)2
Cd3(PO4)2	-14.62	-47.22	-32.60	Cd3(PO4)2
CdSO4	-13.24	-13.34	-0.10	CdSO4
CO2(g)	-1.08	-2.55	-1.47	CO2
Dolomite	0.83	-16.26	-17.08	CaMg(CO3)2
Gypsum	-2.33	-6.91	-4.58	CaSO4:2H2O
H2(g)	-41.18	-44.29	-3.10	H2
H2O(g)	-1.51	-0.00	1.50	H2O

Halite	-3.25	-1.68	1.57	NaCl
Hydroxyapatite	4.15	0.73	-3.42	Ca5(PO4)3OH
O2(g)	-0.92	-3.82	-2.89	O2
Otavite	-2.48	-14.58	-12.10	CdCO3
Sylvite	-4.47	-3.57	0.90	KCl

**For a gas, SI = log10(fugacity). Fugacity = pressure * phi / 1 atm.
For ideal gases, phi = 1.

End of simulation.

Reading input data for simulation 2.

End of Run after 9.955 Seconds.
